# Complexes of Dichlorogermylene with Phosphine/Sulfoxide-Supported Carbone as Ligand †

**DOI:** 10.3390/molecules26072005

**Published:** 2021-04-01

**Authors:** Ugo Authesserre, Sophie Hameury, Aymeric Dajnak, Nathalie Saffon-Merceron, Antoine Baceiredo, David Madec, Eddy Maerten

**Affiliations:** 1 Université de Toulouse, UPS, and CNRS, LHFA UMR 5069, 118 Route de Narbonne, 31062 Toulouse, France; authesserre@chimie.ups-tlse.fr (U.A.); sophie.hameury@univ-poitiers.fr (S.H.); dajnak@chimie.ups-tlse.fr (A.D.); baceiredo@chimie.ups-tlse.fr (A.B.); 2 Université de Poitiers, IC2MP, UMR CNRS 7285, 1 Rue Marcel Doré, CEDEX 9, 86073 Poitiers, France; 3 Université de Toulouse, UPS, and CNRS, ICT UAR2599 118 Route de Narbonne, 31062 Toulouse, France; saffon@chimie.ups-tlse.fr

**Keywords:** carbone, ligand, germylene, coordination, ylide

## Abstract

Due to their remarkable electronic features, recent years have witnessed the emergence of carbones L_2_C, which consist in two donating L ligands coordinating a central carbon atom bearing two lone pairs. In this context, the phosphine/sulfoxide-supported carbone **4** exhibits a strong nucleophilic character, and here, we describe its ability to coordinate dichlorogermylene. Two original stable coordination complexes were obtained and fully characterized in solution and in the solid state by NMR spectroscopy and X-ray diffraction analysis, respectively. At 60 °C, in the presence of **4**, the Ge(II)-complex **5** undergoes a slow isomerization that transforms the bis-ylide ligand into an yldiide.

## 1. Introduction

**Figure 1:**
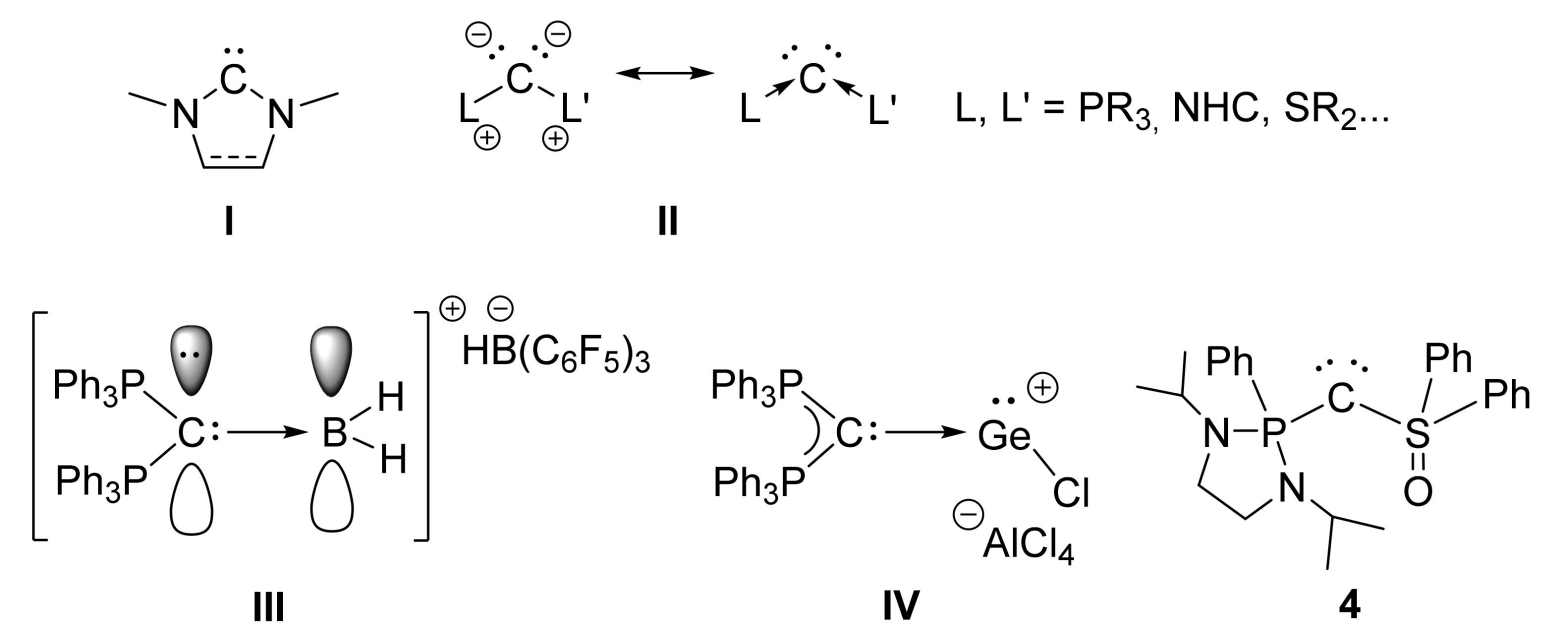
NHC (**I**), carbones (**II)**, dihydrido borenium (**III**) and germyliumylidene (**IV**) stabilized by carbodiphosphoranes and phosphine/sulfoxide-supported carbone **4** (for a better readability, formal charges in (**III**,**IV**) and **4** were omitted).

The rapid development of homogeneous catalysis in the last decades is highly related to the intensive research that was accomplished toward ligand design. Due to their lone pair(s) and their related nucleophilic character, oxygen-, nitrogen-, sulfur- and phosphorus-containing ligands have been dominating in the field for several decades [1]. In the late 1980s, the discovery of the first stable carbenes represented the milestone leading to the emergence of carbon-based ligands (**I**, Figure 1) [2,3]. Indeed, the development of these molecules, containing a divalent C(II) atom bearing a vacant orbital and a lone pair, exhibiting a high σ-donation and a strong binding ability toward transition metals, render them essential tools for catalysis. The corresponding organometallic complexes have been proven to be particularly robust and efficient and offering in numerous catalytic processes a larger scope of reaction [4,5,6,7]. The related carbon(0) species (**II**), also named carbones, bearing two lone pairs on the central carbon atom are a new emerging class of η^1^-carbon ligands. Even though carbodiphosphoranes (**II**, L, L′ = PPh_3_) were discovered in the 1960s by Ramirez [8], these species were at first regarded as two cumulated phosphorus ylide functions on a central carbon atom. It was only in 2006, after the theoretical investigations by Frenking et al. [9,10,11,12], that these molecules were considered as a carbon atom in the zero-oxidation state stabilized by two L-phosphine ligands, in agreement with the description initially used by Kaska in 1973 [13]. Since then, this family of ligands has considerably grown, leading to a large structural diversity of carbones **II** and a better understanding of their behavior [14,15,16,17,18,19,20,21,22,23]. Naturally, owing to the existence of two lone pairs, they are strong σ- and π-donors (two- or four-electron donating ability); they have been used as original ligands for the preparation of organometallic complexes with interesting applications in catalysis [24,25,26,27,28,29].

The strong donor ability of carbones **II** also enables the synthesis and isolation of novel reactive species. For example, Alcarazo et al. took advantage of the two available lone pairs of carbodiphosphoranes to stabilize reactive molecules such as dihydrido borenium cation **III** [30]. In the same vein, several groups have used carbones or strong σ-donating ligands to prepare dichlorogermylene adducts giving access to germyliumylidenes (**IV**) or germylones [31,32,33,34,35,36,37,38]. In this context, we report here the coordination ability of a phosphine/sulfoxide-supported carbone **4** [39] towards dichlorogermylene.

## 2. Results and Discussion

### 2.1. Synthesis

**Scheme 1:**
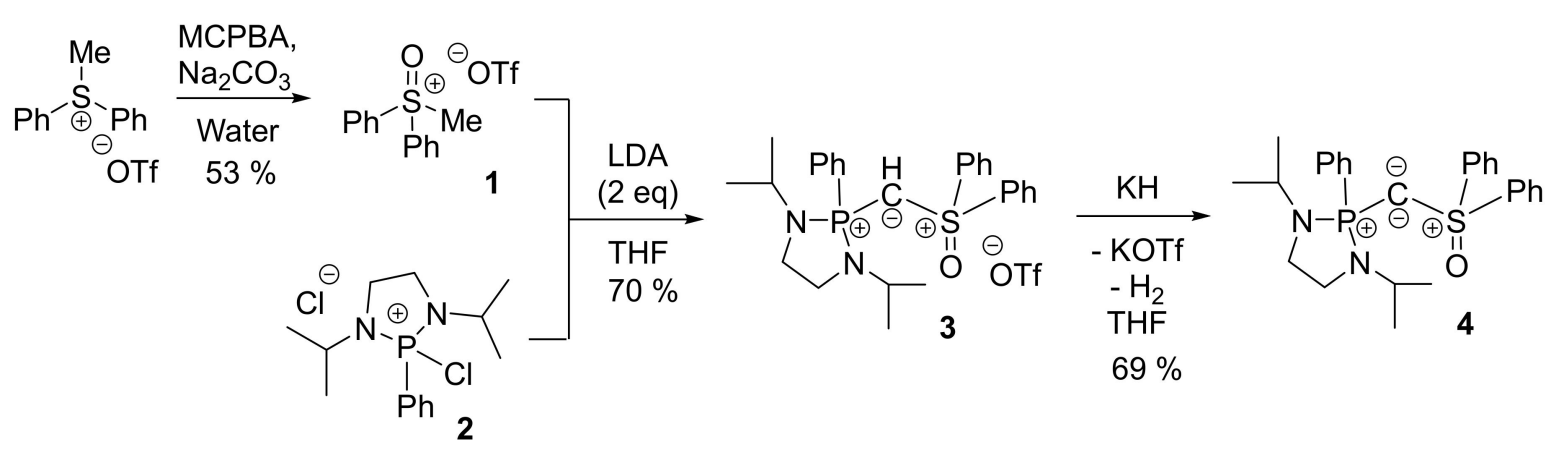
Synthetic path of phosphine/sulfoxide-supported carbone **4**.

For the preparation of the phosphine/sulfoxide-supported carbone **4**, we followed the previously described synthesis [39] but several practical aspects were improved. Indeed, after the complete oxidation of methyldiphenylsulfonium, acidification and filtration of the precipitates (carboxylic acids), the expected methyldiphenylsulfoxonium salt **1** was extracted from the aqueous solution by liquid/liquid extraction using dichloromethane as a solvent (Scheme 1). This extraction avoids the possible thermal degradation of the sulfoxonium salt during the evaporation of water under reduced pressure (if prolonged heating above 50 °C is performed). The yield of this step was improved to 53%, after two successive recrystallizations. The coupling reaction between sulfoxonium salt **1** and chlorophosphonium **2** in the presence of two equivalents of lithium di*iso*propylamide (LDA) was also improved in terms of reaction time (Scheme 1). It was found that heating the reaction mixture up to 60 °C considerably sped up the reaction since a full conversion was reached in 24 h instead of 96 h at room temperature. Protonated precursor **3** was obtained as a white powder upon concentration (70% yield). The final deprotonation was performed in THF solution at RT with potassium hydride (KH) leading to the selective formation of **4**, which was isolated in 69% yield.

### 2.2. Dichlorogermylene Coordination

Previous experimental results and DFT calculations have already established that phosphine/sulfoxide-supported carbone **4** exhibits a strong nucleophilic character [39]. The potential usefulness of **4** as a ligand towards transition metals was demonstrated by selective reactions with several organometallic complexes [Au(I), Rh(I)] [39]. Therefore, its coordinating ability toward GeCl_2_/dioxane should be an interesting approach to access original low-valent germanium derivatives.

**Scheme 2:**
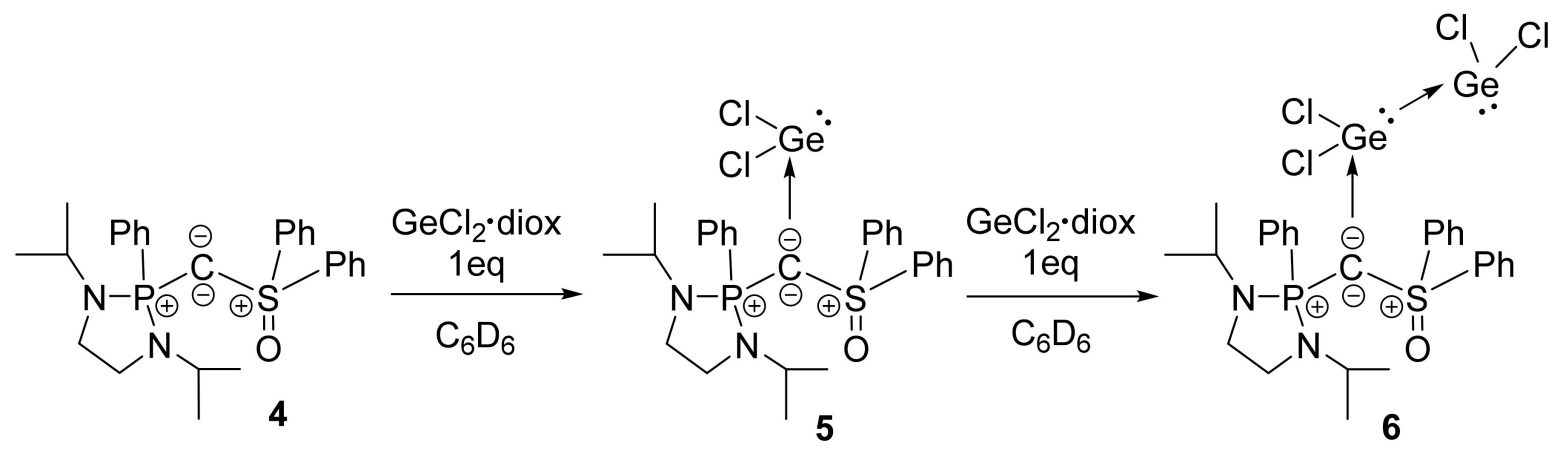
Coordination of phosphine/sulfoxide-supported carbone **4** with one or two equivalents of germylene dichloride.

Ligand **4** reacts immediately with one equivalent of GeCl_2_ dioxane leading to the selective formation of complex **5** which has been isolated as colorless crystals from a saturated solution of CH_2_Cl_2_/pentane (yield 80%, Scheme 2). In comparison with the hexaphenylcarbodiphosphorane analogue [33], complex **5** exhibits a good solubility in common organic solvent and could be fully characterized by NMR spectroscopy. The ^31^P NMR spectrum displays a signal at lower field (δ = 49.5 ppm) compared to that of free ligand **4** (δ = 29.0 ppm), reminiscent of the protonated precursor **3** (δ = 45.0 ppm). In the ^13^C NMR spectrum, the central carbon atom appears as a doublet at δ = 47.0 ppm (J_PC_ = 77.3 Hz). The addition of a second equivalent of GeCl_2_·dioxane to **5**, or the direct use of two equivalents of GeCl_2_·dioxane with **4**, leads to the quantitative formation of bis-germylene complex **6**, which has been isolated in the crystalline form from C_6_D_6_ solution (yield 75%). It has been fully characterized by NMR spectroscopy, and particularly, the ^31^P NMR spectrum indicates a signal at δ = 51.2 ppm and the central carbon atom appears in ^13^C NMR spectrum as a doublet at δ = 46.6 ppm (J_PC_ = 79.1 Hz).

**Figure 2:**
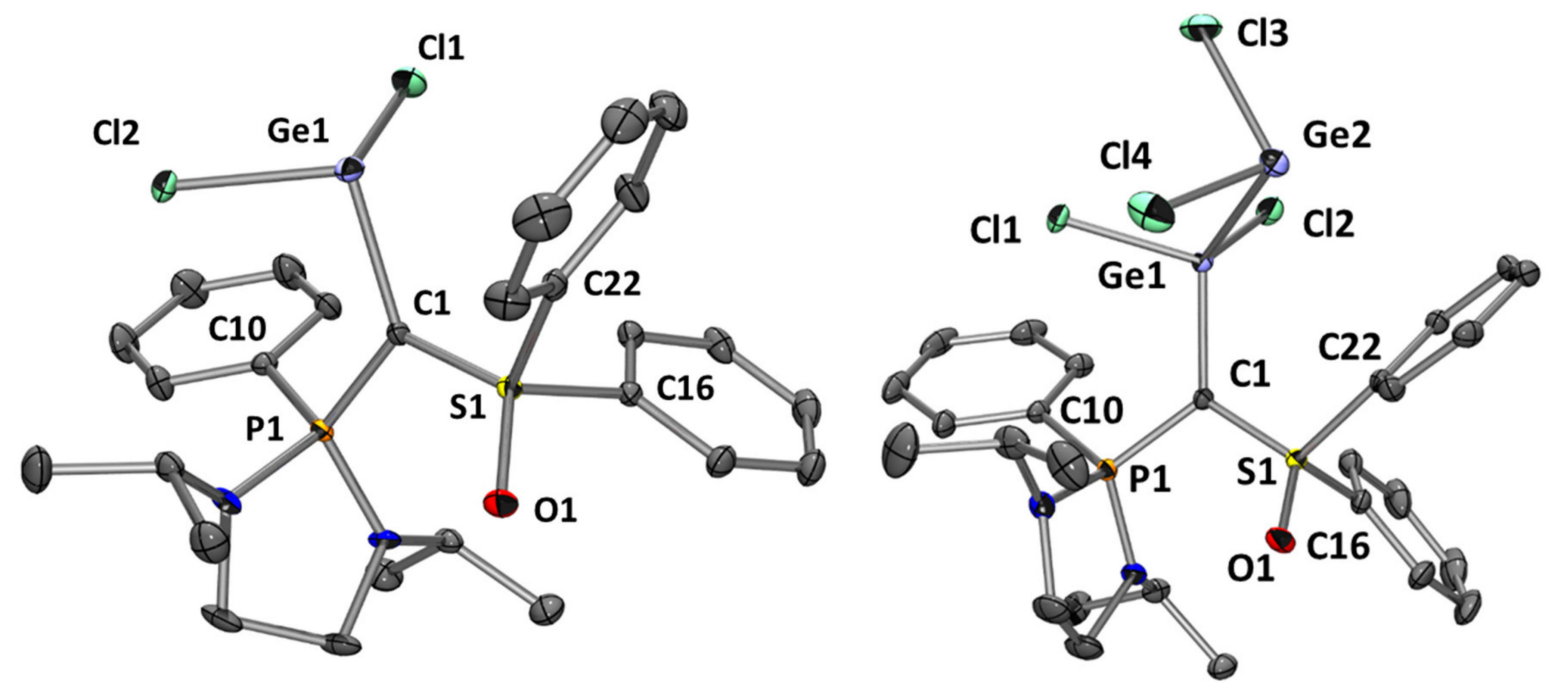
Molecular structures of **5** (**left**) and **6** (**right**). Ellipsoids are drawn at the 30% probability level; hydrogen atoms are omitted for clarity. Selected bond lengths (Å) and angles (°): **5**: C1-Ge1 2.071(2), C1-P1 1.725(2), C1-S1 1.650(2), P1-C10 1.802(3), S1-O1 1.457(2), Ge1-Cl2 2.299(1), Ge1-Cl1 2.331(1), S1-C22 1.780(3), S1-C16 1.784(3). P1-C1-S1 113.73(14), P1-C1-Ge1 128.12(13), Ge1-C1-S1 115.65(13), C1-Ge1-Cl2 96.82(7), Cl2-Ge1-Cl1 95.37(3), Cl1-Ge1-C1 98.81(7); **6**: C1-Ge1 1.980(2), C1-P1 1.748(2), C1-S1 1.665(2), Ge1-Ge2 2.582(1), Ge1-Cl1 2.185(1), Ge1-Cl2 2.245(1), Ge2-Cl4 2.267(1), Ge2-Cl3 2.275(1), P1-C10 1.792(2), S1-O1 1.455(1), S1-C22 1.778(2), S1-C16 1.782(2), P1-C1-S1 112.40(10), P1-C1-Ge1 127.01(9), Ge1-C1-S1 117.30(9), C1-Ge1-Cl1 105.44(5), Cl1-Ge1-Cl2 101.33(2), Cl2-Ge1-Ge2 107.25(2), Cl1-Ge1-Ge2 109.25(2), Ge1-Ge2-Cl4 92.09(2), Cl4-Ge2-Cl3 98.84(3), Ge1-Ge2-Cl3 89.41(2).

Isolated in the crystalline form, the molecular structures of complexes **5** and **6** were confirmed by X-ray diffraction analysis (Figure 2, see Supplementary Materials). The selected geometrical parameters for experimental structures can be found in Table 1.

As expected, the S1-C1 (1.650 Å) and P1-C1 (1.725 Å) bond lengths in **5** are very similar to those observed in the protonated precursor **3** (1.653 Å and 1.719 Å respectively), because of their similar environments. The repulsion between the p_π_-lone pair at the carbon and the one at the Ge atom explains the long C1-Ge1 bond length (2.071 Å). It is slightly longer than the one observed by Alcarazo et al. with carbodiphosphoranes (2.063 Å) but much longer than a typical C-Ge bond (1.95 Å) [40]. The P1-C1-S1 angle decreased significantly upon complexation with GeCl_2_ (from 121° in **3** or **4** to 113.7° in **5**). The introduction of a third heteroatom (Ge) around the carbon, which is less electronegative than P and S, influences the atomic orbital distribution with a pronounced s character toward the C-Ge bond and increased p-character in the C-P and C-S bonds. This phenomenon together with the covalent radius of the germanium atom justify the narrowing of the P1-C1-S1 angle in **5** [41]. Nevertheless, the C1 atom environment remains almost planar (∑°= 357.5°). In **6**, with the coordination of the second GeCl_2_ unit, the previously discussed repulsion disappears, leading to a shortening of the C1-Ge1 bond length to 1.980 Å. The other bond lengths and angles (P1-C1, C1-S1, PCS) remain almost unchanged (Table 1). The dative nature of the Ge1-Ge2 bond is confirmed by its length (2.582 Å), way longer than classical Ge-Ge σ-bonds (2.40–2.50 Å) [42]. The same tendency was observed for carbodiphosphoranes [33] or NHC [43].

**Scheme 3:**
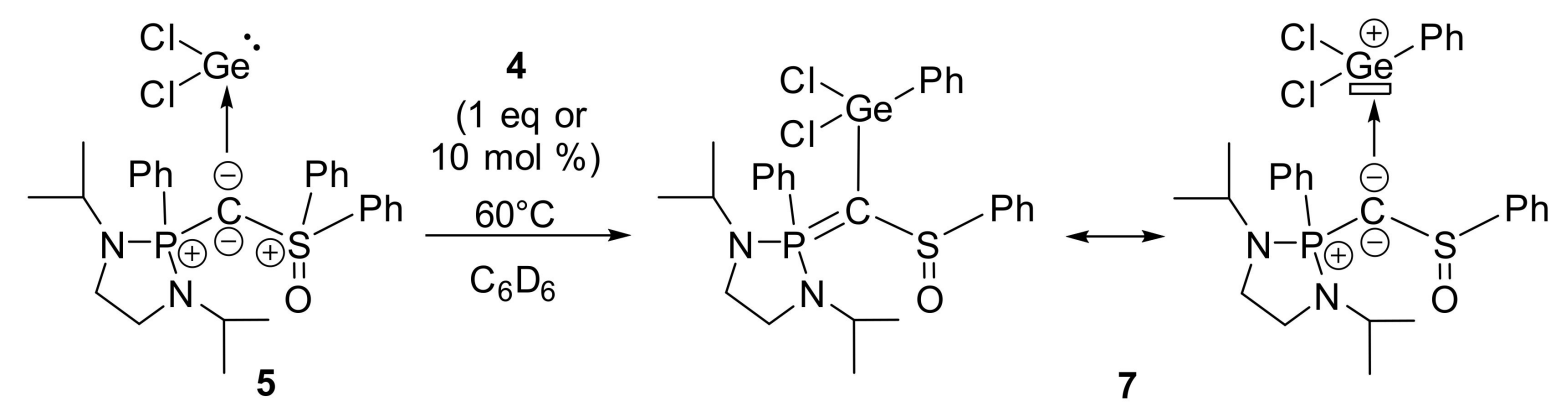
Evolution of **5** in presence of **4** upon heating at 60 °C.

**Figure 3:**
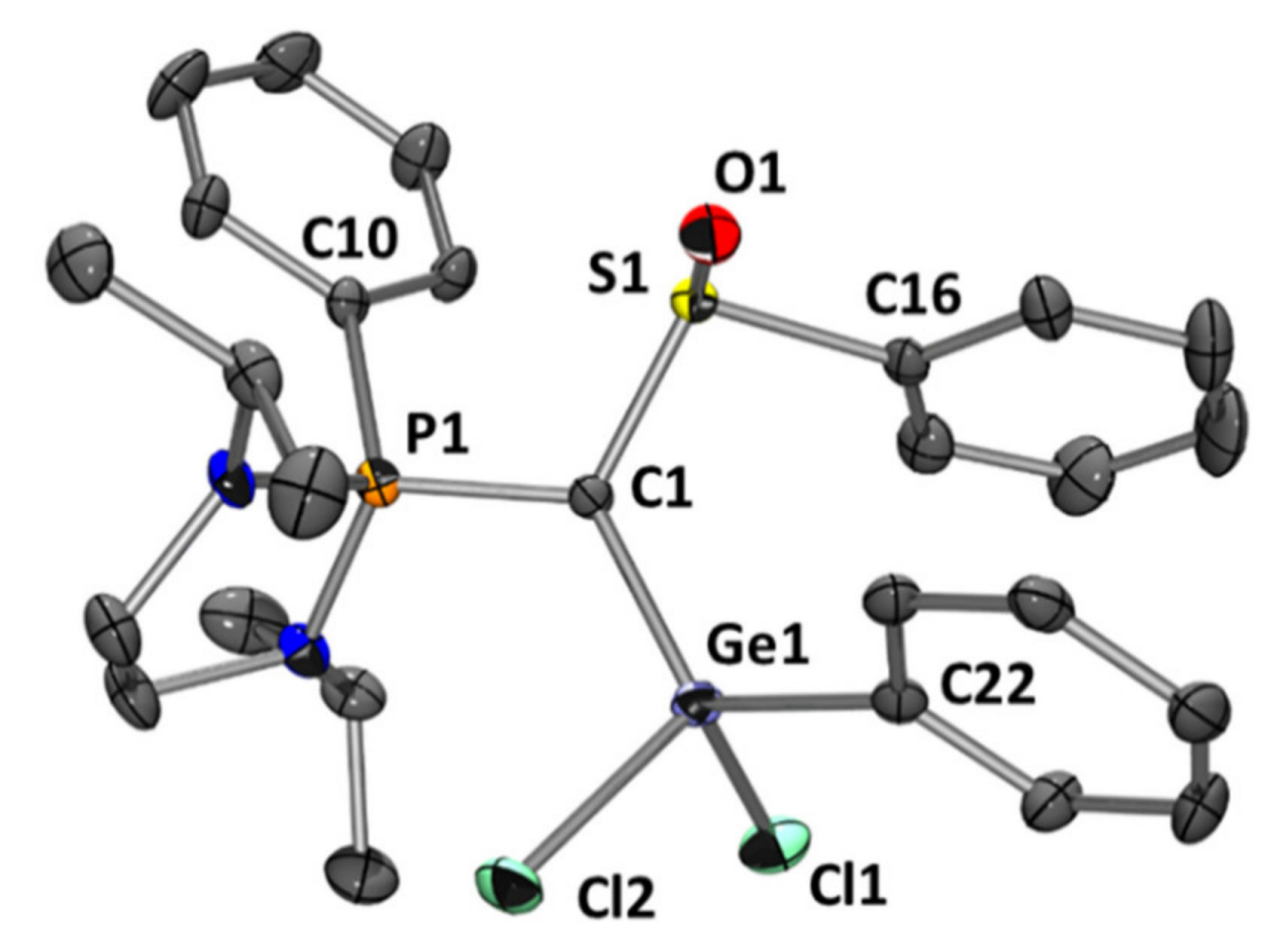
Molecular structure of **7**. Ellipsoids are drawn at the 30% probability level; hydrogen atoms are omitted for clarity. Selected bond lengths (Å) and angles (°): P1-C1 1.707(3), C1-S1 1.744(3), C1-Ge1 1.891(3), Ge1-C22 1.927(3), Ge1-Cl2 2.172(1), Ge1-Cl1 2.173(1), P1-C10 1.805(3), S1-O1 1.499(2), S1-C16 1.800(4), P1-C1-S1 114.48(18), S1-C1-Ge1 120.89(17), Ge1-C1-P1 122.37(19), C1-Ge1-C22 119.52(14), C1-Ge1-Cl2 111.27(10), C22-Ge1-Cl2 104.77(10), C1-Ge1-Cl1 111.72(10), C22-Ge1-Cl1 106.65(11), Cl2-Ge1-Cl1 101.15(4), O1-S1-C1 112.64(15), O1-S1-C16 105.28(16), C1-S1-C16 100.04(16).

Contrary to the carbodiphosphorane analogues, chloride abstraction from **5** using AlCl_3_ or KB(C_6_F_5_)_4_ in order to prepare germyliumylidene derivatives only led to complex mixtures. Nevertheless, when **5** is treated in the presence of one equivalent of **4** at 60 °C for 60 h, we observed the gradual consumption of **5** with the concomitant formation of a new compound that exhibits a signal at δ = 56.3 ppm in ^31^P NMR (this reaction does not occur in the absence of **4**) (Scheme 3). In fact, the process is base-catalyzed, but with 10 mol % of **4,** the reaction time is slower and needs 90 h [Note: catalytic amounts (15 mol %) of alternative Lewis bases such as DMAP or Et_3_N can be used but the reactions are less selective, see Supplementary Materials for more details]. The structure of the new product **7**, determined by X-ray diffraction analysis (Figure 3), involves the 1,3-migration of a phenyl group from the sulfur to the germanium atom. Considering the ligand moiety, this isomerization transforms a bis-ylide (carbone) to an original yldiide [44]. Unfortunately, because of the presence of **4** in the media, product **7** could not be isolated in pure form for complete characterization despite several attempts.

### 2.3. Conclusions

In summary, the excellent coordination ability of the phosphine/sulfoxide-supported carbone ligand **4** allows the preparation of Ge(II)-complex **5**. The strong donation of **4** results in an enriched germylene **5,** which becomes sufficiently nucleophilic to coordinate a second GeCl_2_ unit. The two original and stable coordination complexes **5** and **6** were fully characterized by NMR spectroscopy and X-ray diffraction analysis. Interestingly, the Ge(II)-complex **5** shows an original isomerization in the presence of **4** that transforms the bis-ylide ligand into an yldiide, thanks to a phenyl migration. Efforts are currently underway to extend the diversity of organometallic complexes that can be obtained with **4** to consider its application in catalysis.

## 3. Materials and Methods

### 3.1. General Comments

All manipulations were performed under an inert atmosphere of argon by using standard Schlenk techniques or high-pressure NMR tube techniques. Dry and oxygen-free solvents were used. ^1^H, ^13^C, ^19^F and ^31^P NMR spectra were recorded on Brucker Avance II 300 MHz, Avance III HD 400 MHz and Avance I and II 500 MHz spectrometers (Brucker, Karlsruhe, Germany). Chemical shifts are expressed in parts per million with residual solvent signals as internal reference (^1^H and ^13^C{^1^H}). ^19^F and ^31^P NMR chemical shifts were reported in ppm relative to CFCl_3_ and 85% H_3_PO_4_, respectively. The following abbreviations and their combinations are used: br, broad; s, singlet; d, doublet; t, triplet; q, quartet; hept, heptuplet; m, multiplet. ^1^H and ^13^C resonance signals were attributed by means of 2D COSY, HSQC and HMBC experiments. Mass spectra were recorded on a Hewlett Packard 5989A spectrometer (Hewlett-Packard, Palo Alto, CA, USA). All commercially available reagents were used without further purification otherwise noted. Preparation of diphenylsulfonium, **2** and **4** were prepared following previously reported procedures [39].

### 3.2. Synthesis

Diphenylmethylsulfoxonium triflate **1**: A suspension of diphenylmethylsulfonium triflate (14.96 g, 42.7 mmol, 1 eq.), Na_2_CO_3_ (13.58 g, 128.1 mmol, 3 eq.) and meta-chloroperbenzoic acid (MCPBA) (22.13 g, 128.1 mmol, 3 eq.) in water (400 mL) was stirred at RT for three days. Na_2_CO_3_ (4.53 g, 42.7 mmol, 1 eq.) and MCPBA (7.28 g, 42.7 mmol, 1 eq.) were added and the mixture was stirred at RT for one additional day. A 37% aqueous solution of HCl was added to the solution until pH = 1. White solid was filtrated off and washed with an aqueous solution of HCl at pH = 1 (3 × 20 mL). The product was then extracted with CH_2_Cl_2_ (3 × 20 mL). The solution was dried then the solvent was removed under reduced pressure. The residue was purified by successive crystallizations in acetone/pentane, yielding **1** as colorless crystals (8.30 g, 22.6 mmol, 53%). ^1^H NMR (300 MHz, 298 K, Acetone-*d*_6_): δ = 4.86 (s, 3H, C*H*_3_), 7.91–7.98 (m, 4H, C*H*_ar_), 8.04–8.11 (m, 2H, C*H*_para_), 8.31–8.37 (m, 4H, C*H*_ar_). ^13^C{^1^H} NMR (75 MHz, 298 K, Acetone-*d*_6_): δ = 39.7 (s, *C*H_3_), 128.4 (s, *C*H_ar_), 132.0 (s, *C*H_ar_), 132.9 (s, *C*_ipso_), 138.1 (s, *C*H_para_). The signal of the triflate could not be detected. ^19^F NMR (282 MHz, 298 K, DMSO-*d_6_)* δ = −77.7 (s). Mp = 157 °C. HRMS (ES +): m/z [M]^+^ calculated for C_13_H_13_OS = 217.0687, found = 217.0690.

Protonated phosphine/sulfoxide precursor **3**: In situ prepared LDA (18.2 mmol, 2 eq.) in THF (20 mL) was added dropwise to a suspension of **1** (4 g, 10.9 mmol, 1.2 eq.) in THF (100 mL) at −80 °C and the reaction was stirred at this temperature for 2 h. A suspension of **2** (2.92 g, 9.1 mmol, 1 eq.) in THF (50 mL) was then added dropwise to the solution at −80 °C. The solution was allowed to warm up to RT then heated at 60 °C for two days. The solution was concentrated (2/3 of the solvent removed) and a white precipitate was observed. The solvent was filtered off and the white solid was washed with small volumes of THF (4 × 2 mL). The remaining solid was extracted in CH_2_Cl_2_ then dried under a high vacuum. Ylide **3** was obtained as a white powder (3.9 g, 6.37 mmol, 70%). ^31^P{^1^H} NMR (202 MHz, 298 K, CDCl_3_): δ = 45.0 ppm (s). ^1^H NMR (300 MHz, 298 K, CDCl_3_): δ = 0.76 (d, *J*_HH_ = 6.5 Hz, 12H, C*H_3i_*_Pr_), 3.08–3.24 (m, 4H, C*H_2_*), 3.40 (hept, *J*_HH_ = 6.5 Hz, 1H, C*H_i_*_Pr_), 3.43 (hept, *J*_HH_ = 6.5 Hz, 1H, C*H_i_*_Pr_), 3.80 (d, *J*_PH_ = 13.7 Hz, 1H, PC*H*S), 7.57 (m, 9H, C*H*_ar_), 8.05–8.16 (m, 2H, C*H*_ar_), 8.18–8.25 (m, 4H, C*H*_ar_). ^13^C{^1^H} NMR (75 MHz, 298 K, CDCl_3_): δ = 19.9–20.1 (m, *C*H_3*iPr*_), 31.6 (d, *J*_CP_ = 137.2 Hz, P*C*HS), 39.1 (d, *J*_PC_ = 8.6 Hz, *C*H_2_), 45.1 (d, *J*_PC_ = 5.7 Hz, *C*H*_i_*_Pr_), 121.2 (q*, J*_CF_ = 318.9 Hz, *C*F_3_), 126.7 (d, *J*_PC_ = 121.1 Hz, *C*_ipso P side_), 127.1 (s, *C*H_ar_), 130.0 (d, *J*_PC_ = 13.7 Hz, *C*H_ar_), 130.4 (s, *C*H_ar_), 132.8 (d, *J*_PC_ = 11.7 Hz, *C*H_ar_), 134.4 (s, *C*H_ar_), 134.5 (d, *J*_PC_ = 3.1 Hz, *C*H_ar_), 140.4 ppm (d, *J*_PC_ = 2.4 Hz, *C*_ipso S side_). ^19^F NMR (282 MHz, 298 K CDCl_3_): δ = −78.1 (s). HRMS (ES +): *m*/*z* [M]^+^ calculated for C_27_H_34_ON_2_PS = 465.2130, found = 465.2133. The elemental analysis calculated (%) for C_28_H_34_F_3_N_2_O_4_PS_2_: C 54.71, H 5.58, N 4.56; found: C 54.63, H 5.47, N 4.51.

Phosphine/sulfoxide-carbone-GeCl_2_ complex **5**: To a solution of **4** (37.1 mg, 0.08 mmol, 1 eq.) in C_6_D_6_ (0.6 mL), GeCl_2_•dioxane (9 mg, 0.08 mmol, 1 eq.) was added. The reaction is complete after a few minutes. C_6_D_6_ was removed under reduced pressure and the residue afforded crystals (38.9 mg, 0.064 mmol, 80% yield) from a saturated solution of CH_2_Cl_2_/pentane. ^31^P {^1^H} NMR (202 MHz, 298 K, C_6_D_6_): δ = 49.5 ppm (s). ^31^P {^1^H} NMR (162 MHz, 298 K, CD_2_Cl_2_): δ = 50.9 ppm (s). ^1^H NMR (500 MHz, 298 K, CD_2_Cl_2_): δ = 0.70 (d, *J*_HH_ = 6.5 Hz, 6H, C*H_3i_*_Pr_), 0.81 (d, *J*_HH_ = 6.5 Hz, 6H, C*H_3i_*_Pr_), 3.08–3.18 (m, 2H, C*H_2_*), 3.19–3.28 (m, 2H, C*H_2_*), 3.58–3.69 (m, 2H, C*H_i_*_Pr_), 7.59–7.71 (m, 9H, C*H*_ar_), 8.07–8.14 (m, 6H, C*H*_ar_). ^13^C{^1^H} NMR (126 MHz, 298 K, CD_2_Cl_2_): δ = 19.9 (m, *C*H_3*i*Pr_), 39.3 (d, *J*_PC_ = 8.9 Hz, *C*H_2_), 46.0 (d, *J*_PC_ = 4.6 Hz, *C*H*_i_*_Pr_), 47.0 (d, *J*_PC_ = 77.3 Hz, P*C*S), 129.1 (s, CH_ar_) 129.9 (d, *J*_PC_ = 125.5 Hz, *C*_ipso P side_), 130.1 (d, *J*_PC_ = 13.9 Hz *C*H_ar_), 130.3 (s, *C*H_ar_) 132.9 (d, *J*_PC_ = 11.8 Hz, *C*H_ar_), 134.2 (d, *J*_PC_ = 3.1 Hz, *C*H_ar_), 134.7(s, *C*H_ar_), 140.7 (d, *J*_PC_ = 3.1 Hz, *C*_ipso S side_). HRMS (DCI-CH_4_): *m*/*z* [M + H]^+^ calcd for C_27_H_34_Cl_2_GeN_2_OPS: 609.0713; found: 609.0715.

Phosphine/sulfoxide-carbone-GeCl_2-_GeCl_2_ complex **6**: To a solution of **4** (37.1 mg, 0.08 mmol, 1 eq.) in C_6_D_6_ (0.6 mL), GeCl_2_•dioxane (18 mg, 0.16 mmol, 2 eq.) was added. Product **6** directly crystallized in C_6_D_6_ (45.0 mg, 0.06 mmol, 75% yield). ^31^P {^1^H} NMR (202 MHz, 298 K, C_6_D_6_): δ = 51.2 ppm (s). ^31^P {^1^H} NMR (202 MHz, 298 K, CD_2_Cl_2_): δ = 51.2 ppm (s). ^1^H NMR (300 MHz, 298 K, CD_2_Cl_2_): δ = 0.70 (d, *J*_HH_ = 6.4 Hz, 6H, C*H_3i_*_Pr_), 0.82 (d, *J*_HH_ = 6.5 Hz, 6H, C*H_3i_*_Pr_), 3.09–3.18 (m, 2H, C*H_2_*), 3.18–3.28 (m, 2H, C*H_2_*), 3.60–3.70 (m, 2H, C*H_i_*_Pr_), 7.60–7.71 (m, 9H, C*H*_ar_), 8.06–8.13 (m, 3H, 6C*H*_ar_). ^13^C{^1^H} NMR (126 MHz, 298 K, CD_2_Cl_2_): δ = 19.7–20.3 (m, *C*H_3*i*Pr_), 39.3 (d, *J*_PC_ = 9.2 Hz, *C*H_2_), 46.1 (d, *J*_PC_ = 5.3 Hz, *C*H*_i_*_Pr_), 46.6 (d, *J_PC_* = 79.1 Hz, P*C*S), 129.1 (s, *C*H_ar_), 129.7 (d, *J_PC_* = 125.0 Hz, *C*_ipso_ deduced from J-Mod), 130.2 (d, *J*_PC_ = 13.9 Hz, *C*H_ar_), 130.4 (s, *C*H_ar_), 132.8 (d, *J*_PC_ = 11.8 Hz, *C*H_ar_), 134.4 (d, *J*_PC_ = 3.1 Hz, *C*H_ar_), 134.8 (s, CH_ar_), 140.5 (d, *J_PC_* = 2.95 Hz, *C*_ipso S side_). HRMS for **6** did not afford satisfactory results.

Isomer **7**: To a solution of **5** (48.6 mg, 0.08 mmol, 1 eq.) in C_6_D_6_ (0.6 mL), one equivalent of **4**, 37.1 mg, 0.08 mmol, 1 eq.) was added. The reaction was heated at 60 °C until complete consumption of **5** (after 60 h). Crystals of **7** could be obtained from the crude mixture. Unfortunately, despite several attempts, **7** was only analyzed by spectroscopy as a mixture. The spectroscopic data were extracted from a mixture containing **7** and **4** in a 51% to 45% ratio (4% of protonated phosphine/sulfoxide precursor **3**). The reaction can also be performed with 10 mol% of **4** (3.7 mg, 0.008 mmol). ^31^P {^1^H} NMR (121 MHz, 298 K, C_6_D_6_): δ = 56.3 ppm (s). ^1^H NMR (300 MHz, 298 K, C_6_D_6_): δ = 0.45 (d, *J*_HH_ = 6.5 Hz, 3H, C*H_3i_*_Pr_), 0.83 (d, *J*_HH_ = 6.6 Hz, 3H, C*H_3i_*_Pr_),1.34–1.50 (m, 6H, C*H_3i_*_Pr_), 2.40–2.70 (m, 2H, C*H_2_*), 3.05–3.15 (m, 2H, C*H_2_*), 4.10–4.40 (m, 2H, C*H_i_*_Pr_), 6.75–7.25 (m, 9H, C*H*_ar_), 7.75–7.85 (m, 2H, C*H*ar), 7.85–8.15 (m, 4H, C*H*_ar_). ^13^C{^1^H} NMR (126 MHz, 298 K, C_6_D_6_): δ = 20.6 (d, *J*_PC_ = 2.4 Hz, *C*H_3*i*Pr_), 20.7 (d, *J*_PC_ = 5.7 Hz, *C*H_3*i*Pr_), 21.7 (d, *J*_PC_ = 6.1 Hz, *C*H_3*i*Pr_), 38.0 (d, *J*_PC_ = 12.3 Hz, *C*H_2_), 38.1 (d, *J*_PC_ = 12.7 Hz, *C*H_2_), 44.5 (d, *J*_PC_ = 7.4 Hz, *C*H*_i_*_Pr_), 44.9 (d, *J*_PC_ = 5.0 Hz, *C*H*_i_*_Pr_), 59.1 (d, *J_PC_* = 121.5 Hz, P*C*S), 127.2 (s, *C*H_ar_), 128.0 (s, *C*H_ar_), 128.3 (s, *C*H_ar_), 128.7 (d, *J_PC_* = 12.1 Hz), 128.9 (s, CH_ar_), 130.2 (d, *J_PC_* = 109.6 Hz *C*_ipso P side_), 130.3 (s, *C*H_ar_), 132.6 (d, *J*_PC_ = 2.9 Hz, *C*H_ar_), 133.4 (s, *C*H_ar_), 133.8 (d, *J*_PC_ = 10.3 Hz, *C*H_ar_), 140.4 (d, *J_PC_* = 6.2 Hz, *C*_ipso S side_), 147.8 (d, *J_PC_* = 21.9 Hz, *C*_ipso Ge side_). HRMS (DCI-CH_4_): *m*/*z* [M + H]^+^ calcd for C_27_H_34_Cl_2_GeN_2_OPS: 609.0713; found: 609.0721.

### 3.3. X-ray Data

The data of the structures for **5**, **6** and **7** were collected at 193 K on a Bruker-AXS APEX II CCD Quazar diffractometer (**7**) equipped with a 30 W air-cooled microfocus source or on a Brucker-AXS D8-Venture diffractometer (**5** and **6**) equipped with a CMOS area detector with MoKα radiation (wavelength = 0.71073 Å) by using phi- and omega-scans. The data were integrated with SAINT, and an empirical absorption correction with SADABS was applied [45]. The structures were solved using an intrinsic phasing method (ShelXT) [46] and refined using the least–squares method on *F*^2^ (ShelXL-2014) [47]. All non-H atoms were treated anisotropically. All H atoms attached to C atoms were fixed geometrically and treated as riding on their parent atoms with C-H = 0.95 Å (aromatic), 0.98 Å (CH_3_), 0.99 Å (CH_2_) or 1.0 Å (CH) with U_iso_(H) = 1.2U_eq_(CH, CH_2_) or U_iso_(H) = 1.5U_eq_(CH_3_).

Supplementary crystallographic data for CCDC-2068304 (**5**), CCDC-2068305 (**6**), CCDC-2068306 (**7**) can be obtained free of charge from The Cambridge Crystallographic Data Centre via https://www.ccdc.cam.ac.uk/structures/ accessed on 25 March 2021.

## Figures and Tables

**Table 1:** Selected geometrical parameters for **3**, **4**, **5** and **6** ^1^.

	3	4	5	6
P1-C1	1.719(15)	1.800(1)	1.725(2)	1.748(2)
S1-C1	1.653(13)	1.593(1)	1.650(2)	1.665(2)
P1-C1-S1	120.98(9)	120.74(8)	113.73(14)	112.40(10)
C1-Ge1	-	-	2.071(2)	1.980(2)
Ge1-Ge2	-	-	-	2.582(1)

^1^ Bond lengths in Å and angle in deg.

## Data Availability

Not applicable.

## Supplementary Materials

The following are available online. NMR spectra and crystallographic data.

## Sample Availability

Samples of the compounds are not available from the authors.
