# Supplementary material for: Complexes of Dichlorogermylene with Phosphine/Sulfoxide-Supported Carbone as Ligand
Source: Molecules. 2021 Apr 1;26(7):2005. doi: 10.3390/molecules26072005 (PMC8037485; doi:10.3390/molecules26072005)
Supplement: Supplementary file 1 [file molecules-26-02005-s001.zip › SI_-_Molecules-Final-revised.pdf]

Supporting information for

Complexes of Dichlorogermylene with Phosphine/Sulfoxide-Supported Carbone as Ligand.

Ugo Authesserre [1], Sophie Hameury [1,2], Aymeric Dajnak [1], Nathalie Saffon-Merceron [3], Antoine Baceiredo [1], David Madec [1,*], and Eddy Maerten [1,*]

[1] Université de Toulouse, UPS, and CNRS, LHFA UMR 5069, 118 route de Narbonne, 31062 Toulouse (France)
[2] Université de Poitiers, IC2MP, UMR CNRS 7285, 1 rue Marcel Doré, 86073 Poitiers Cedex 9 (France)
[3] Université de Toulouse, UPS, and CNRS, ICT UAR2599 118 route de Narbonne, 31062 Toulouse (France)
* Correspondence : madec@chimie.ups-tlse.fr ; maerten@chimie.ups-tlse.fr

	Page
**Table of contents**	**S2**
**Selected spectroscopic data**	**S3**
**X-ray analysis**	**S10**

Selected spectroscopic data

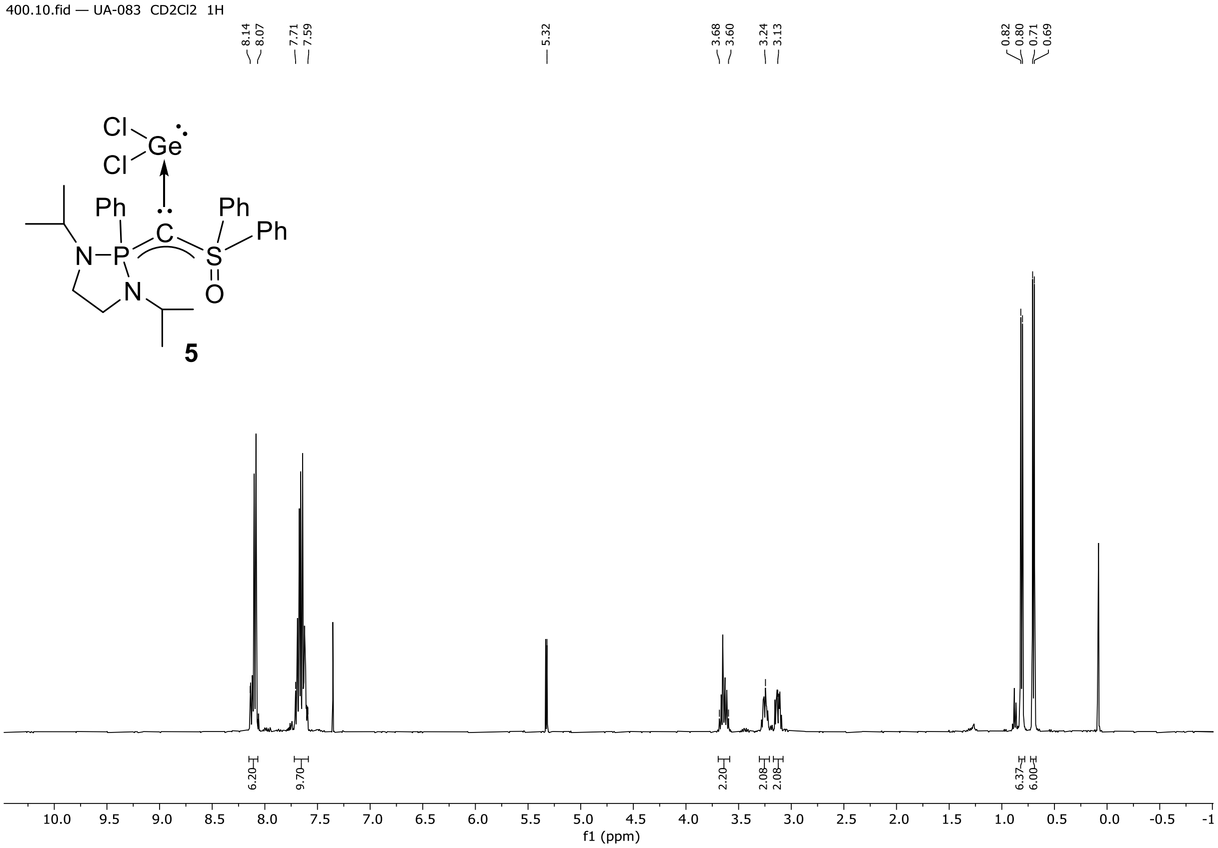

Figure S1: ^{1}H NMR (400 MHz, CD_2Cl_2)

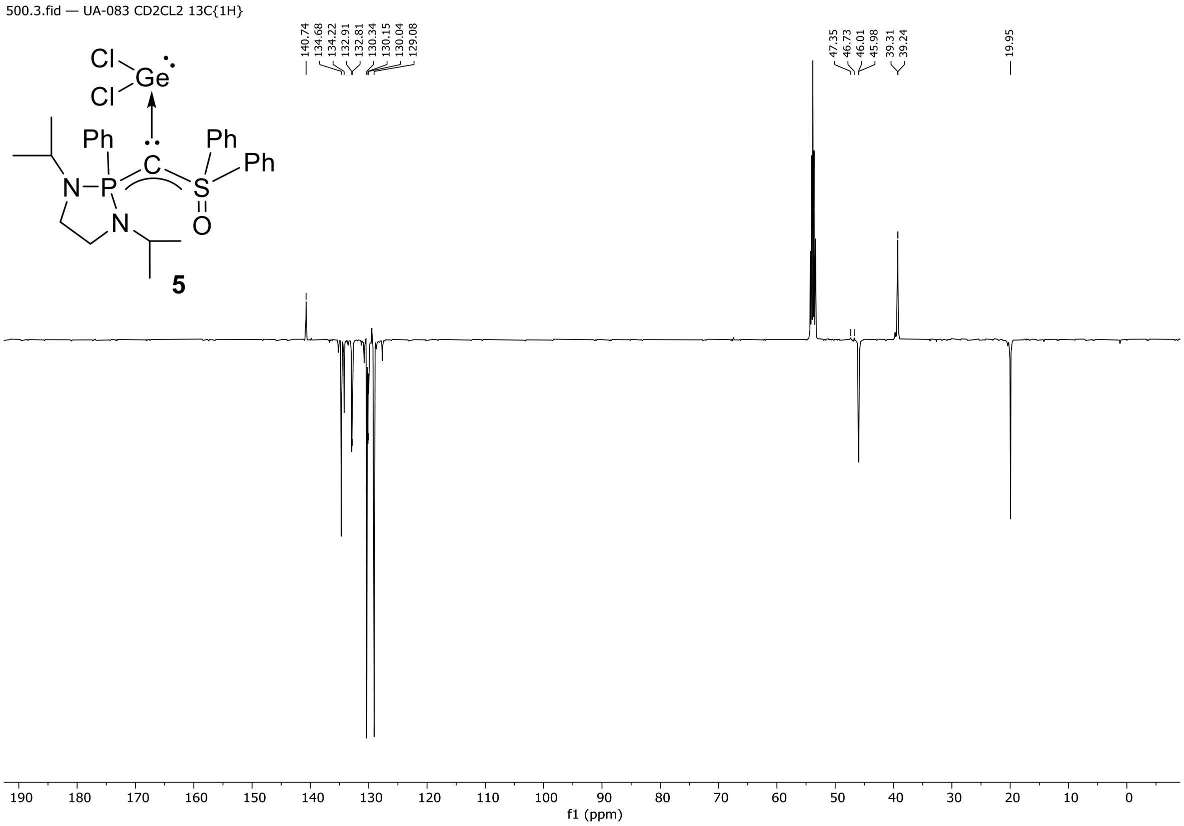

Figure S2: J-mod $^{13}C\{^{1}H\}$ NMR (125 MHz, CD_2Cl_2)

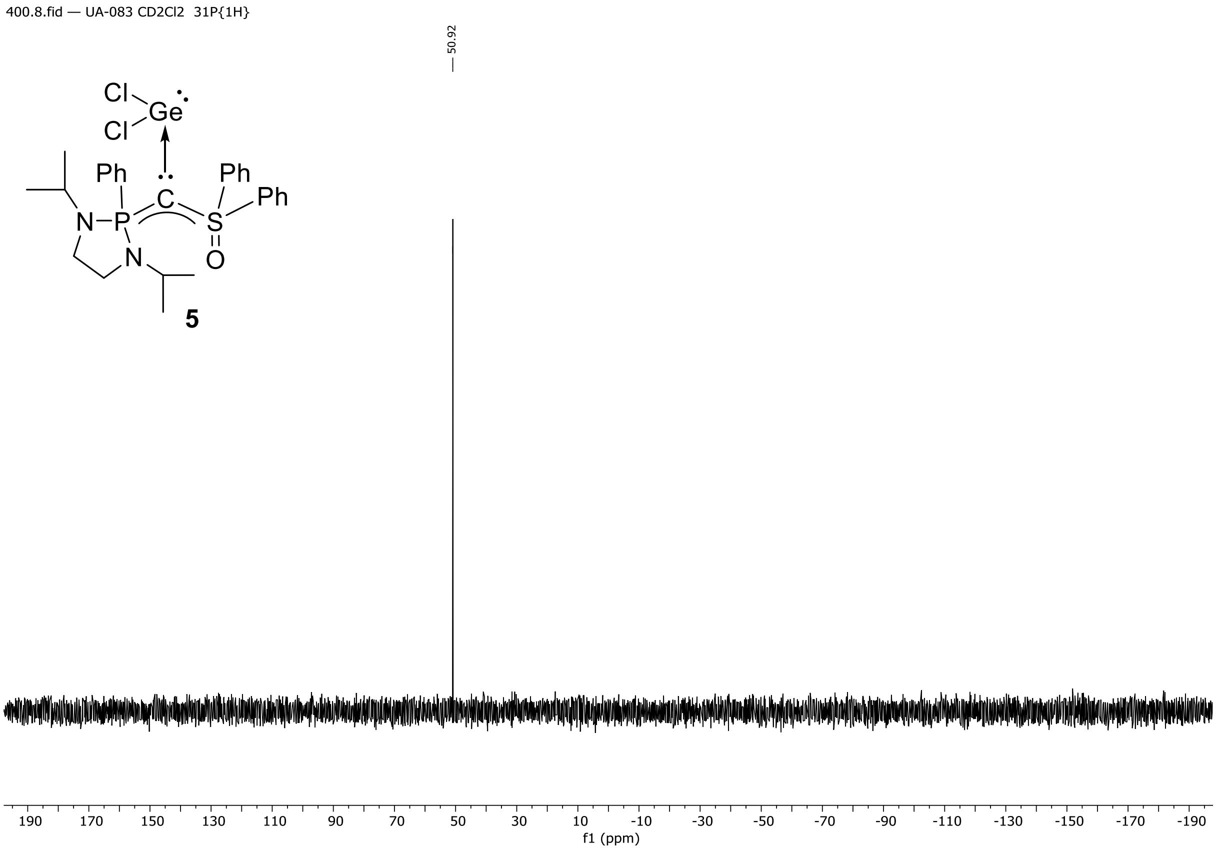

Figure S3: $^{31}P\{^{1}H\}$ NMR (161 MHz, CD_2Cl_2)

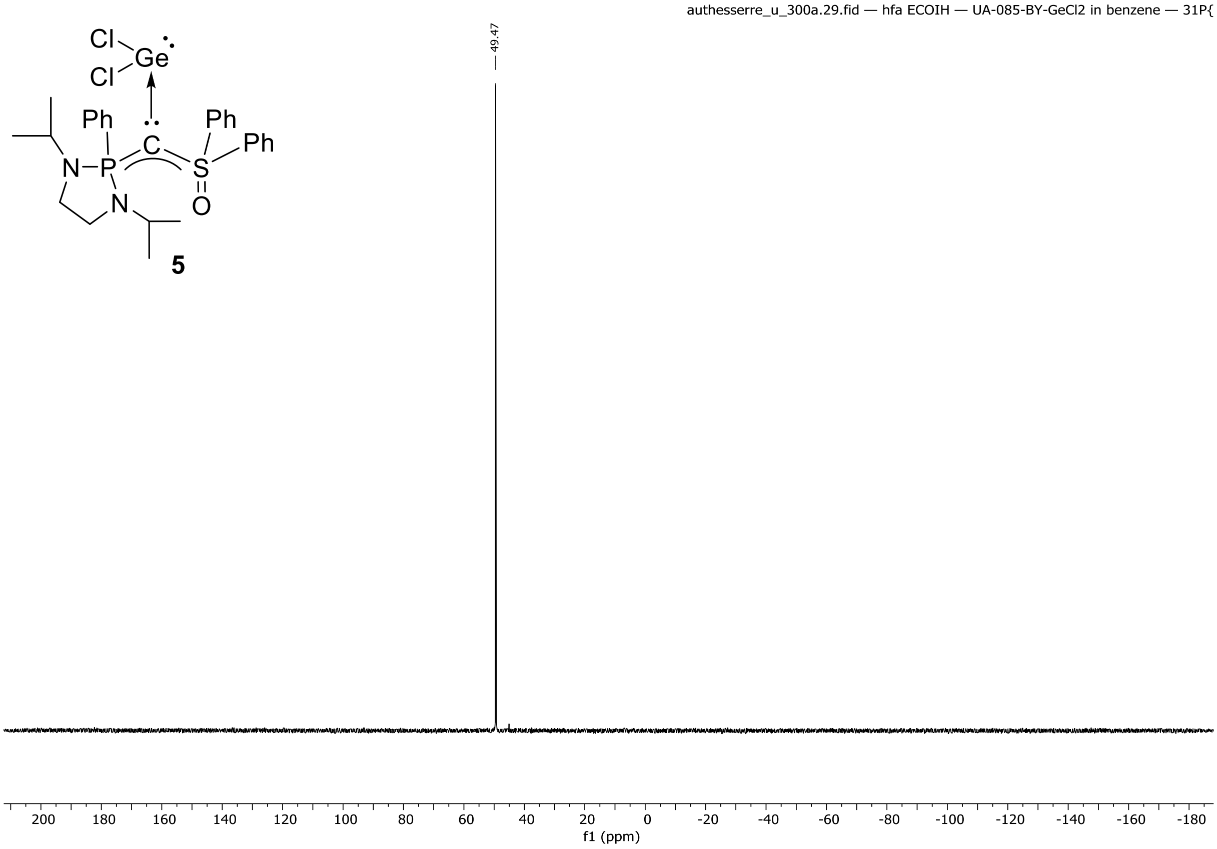

Figure S4: $^{31}P\{^{1}H\}$ NMR (161 MHz, C_6D_6)

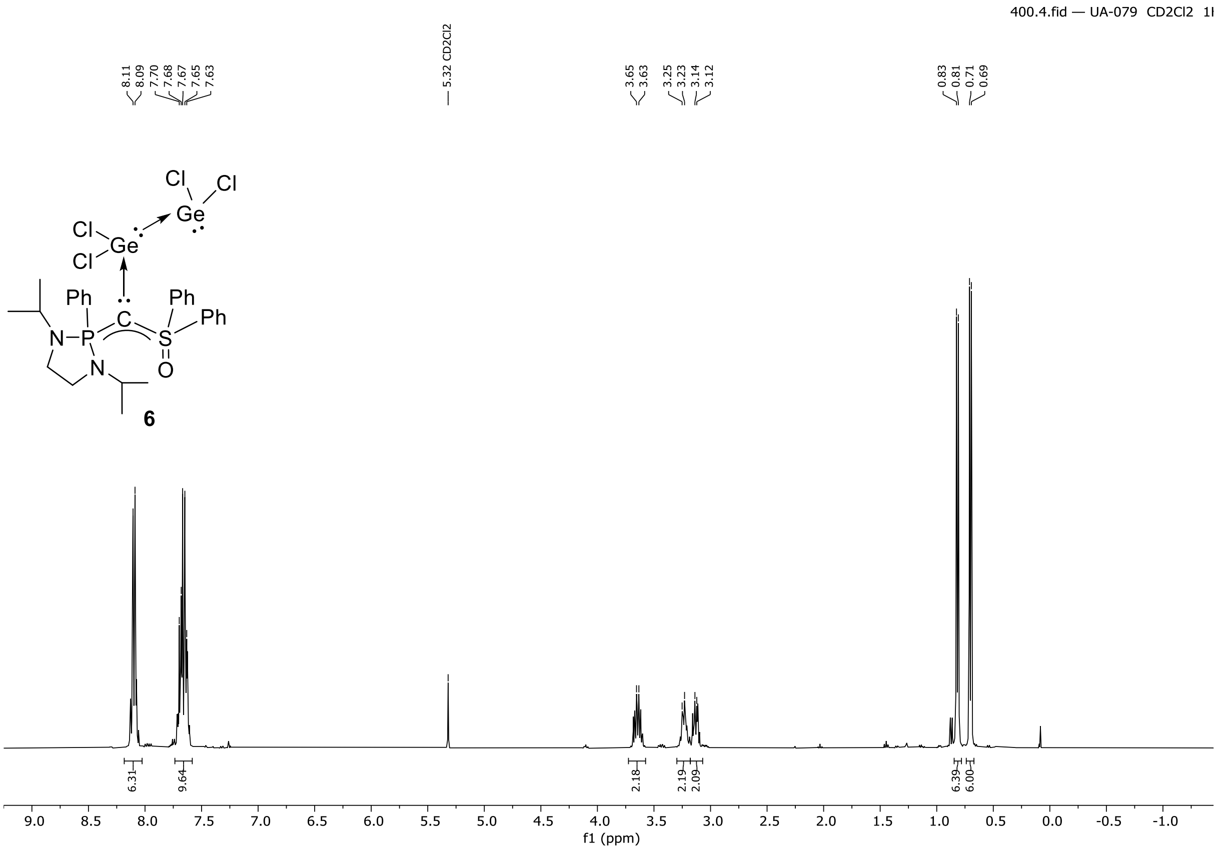

Figure S5: ^{1}H NMR (400 MHz, CD_2Cl_2)

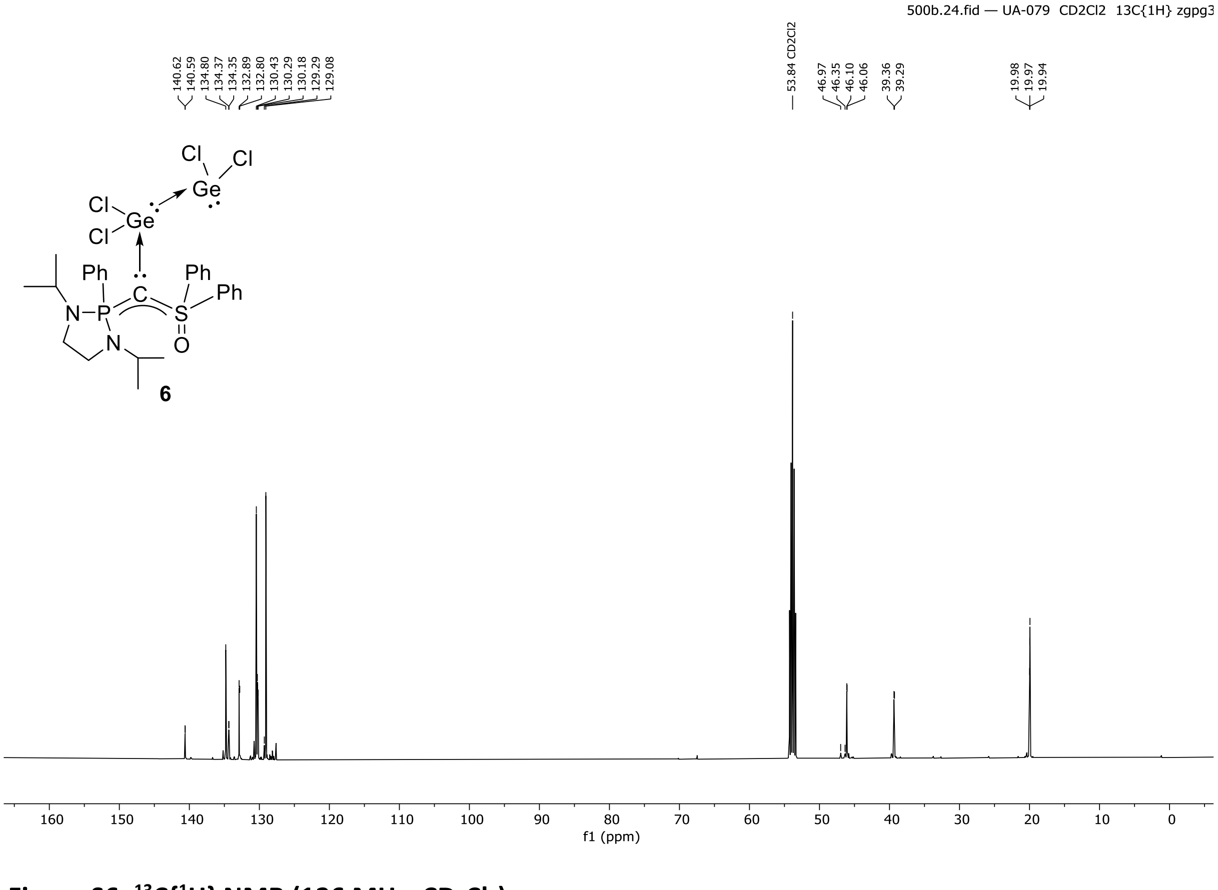

Figure S6: $^{13}C\{^1H\}$ NMR (126 MHz, CD_2Cl_2)

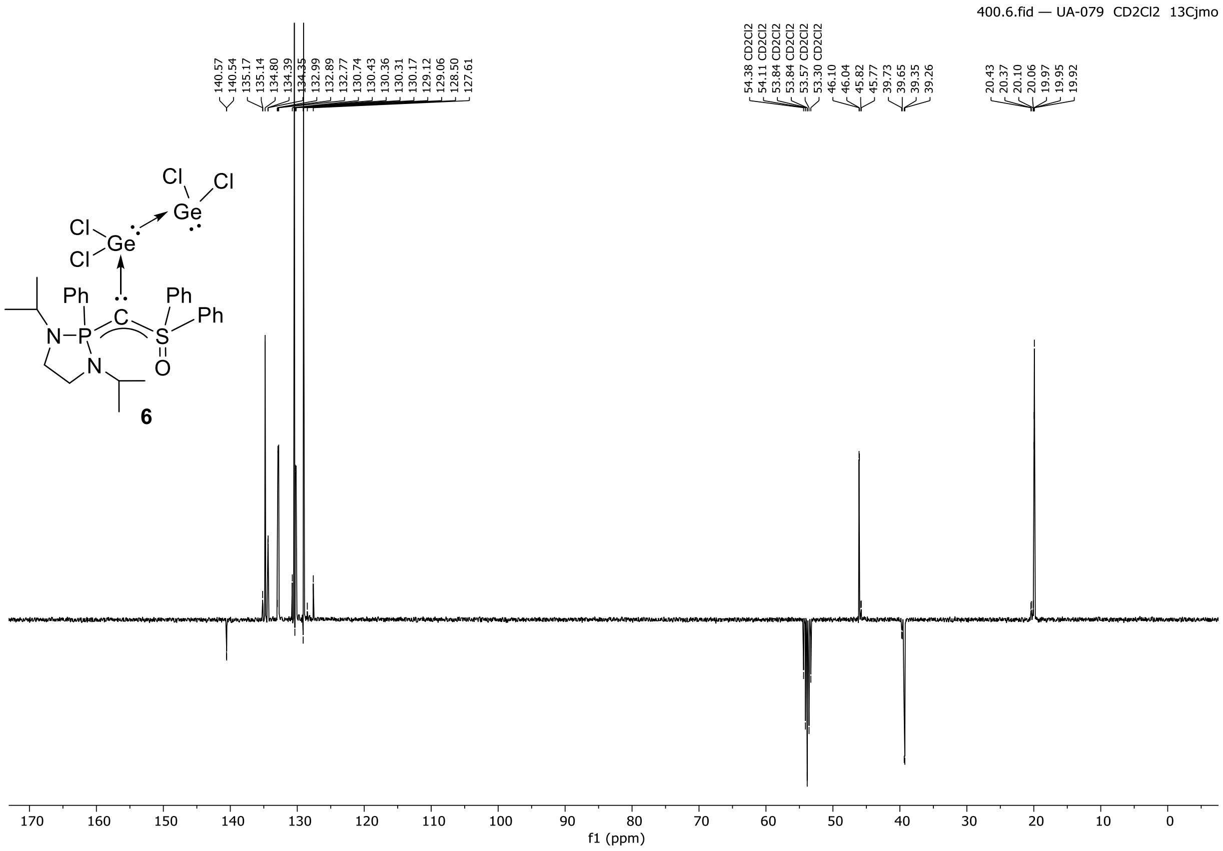

Figure S7: J-Mod$^{13}C\{^1H\}$ NMR (101 MHz, CD_2Cl_2)

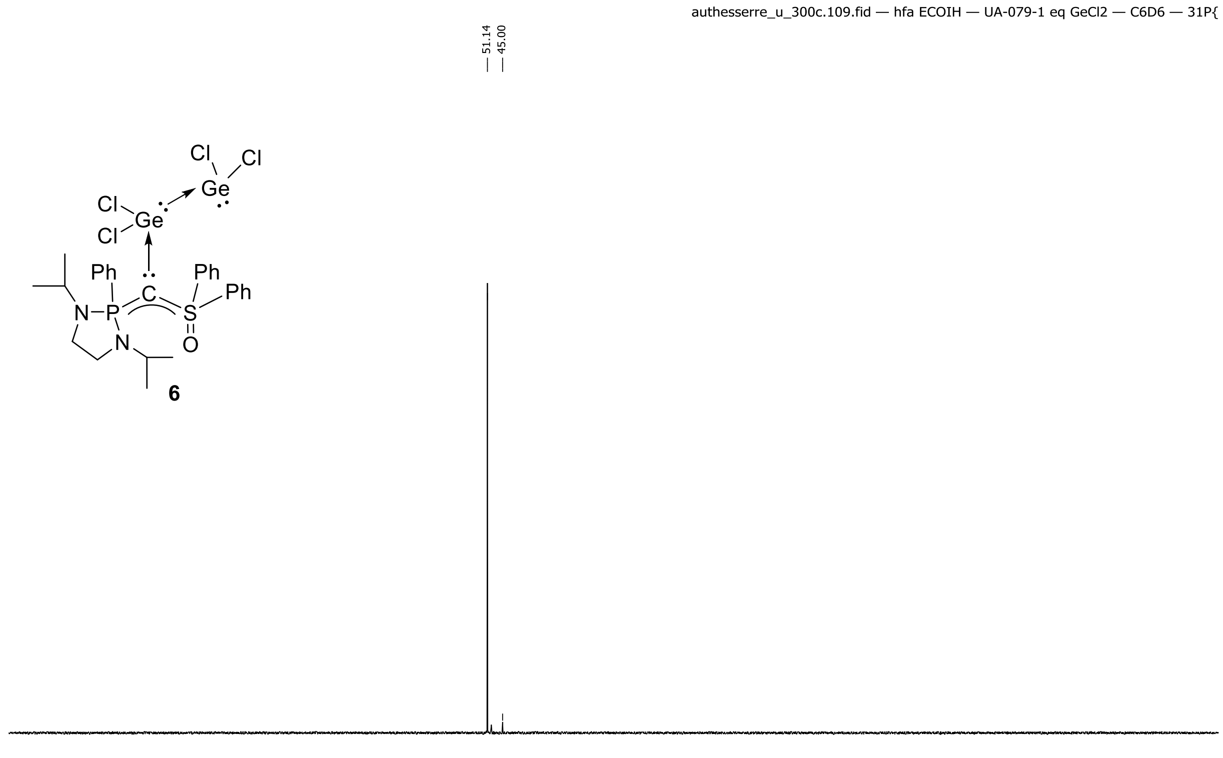

Figure S8: $^{31}P\{^1H\}$ NMR (121 MHz, C_6D_6)

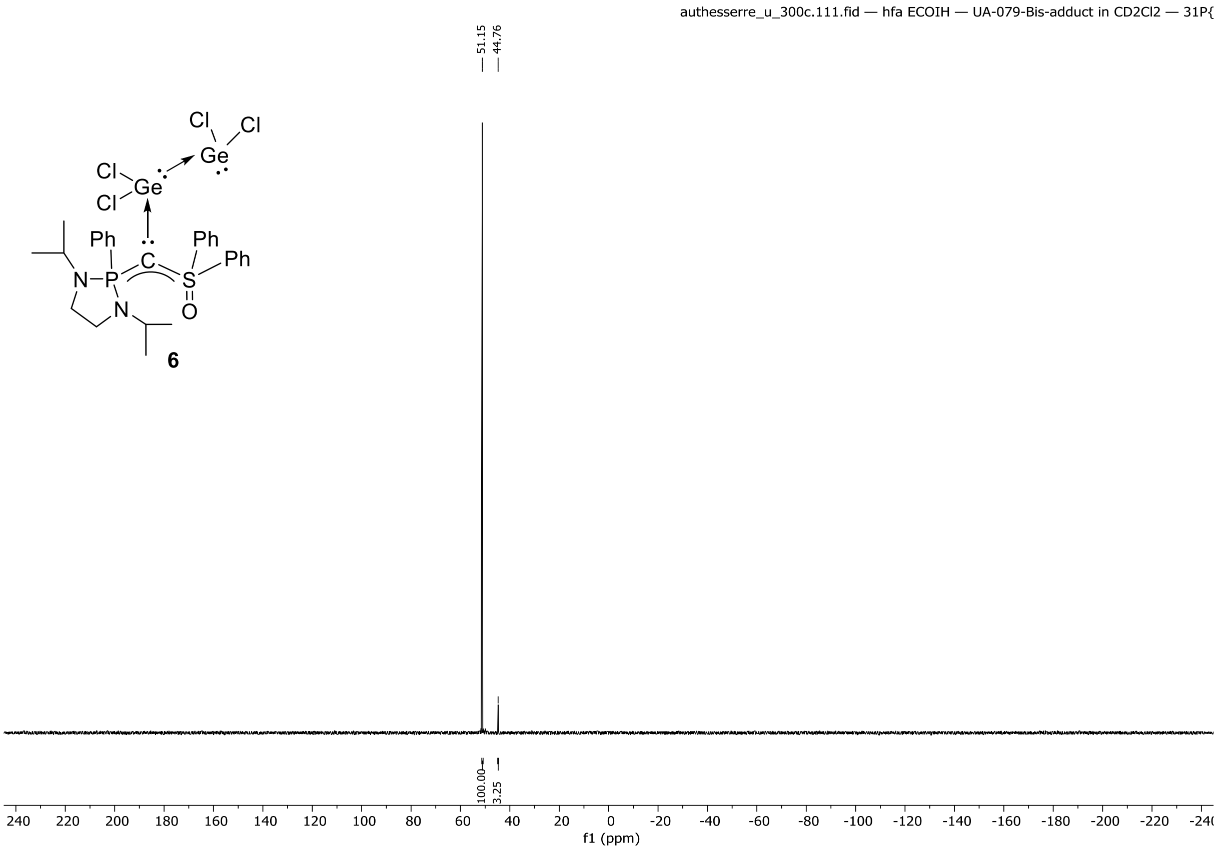

Figure S9: $^{31}P\{^{1}H\}$ NMR (121 MHz, CD_2Cl_2)

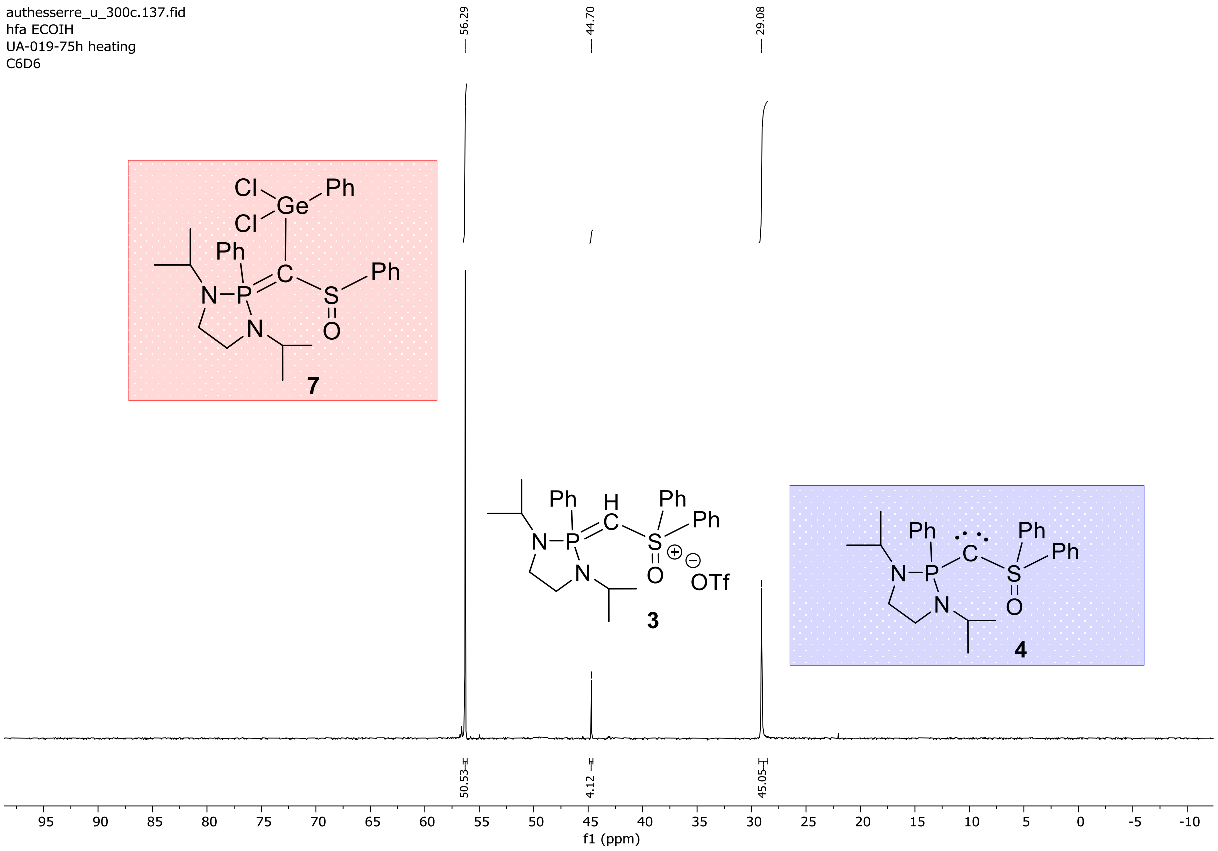

Figure S10: $^{31}P\{^{1}H\}$ NMR (121 MHz, C_6D_6)

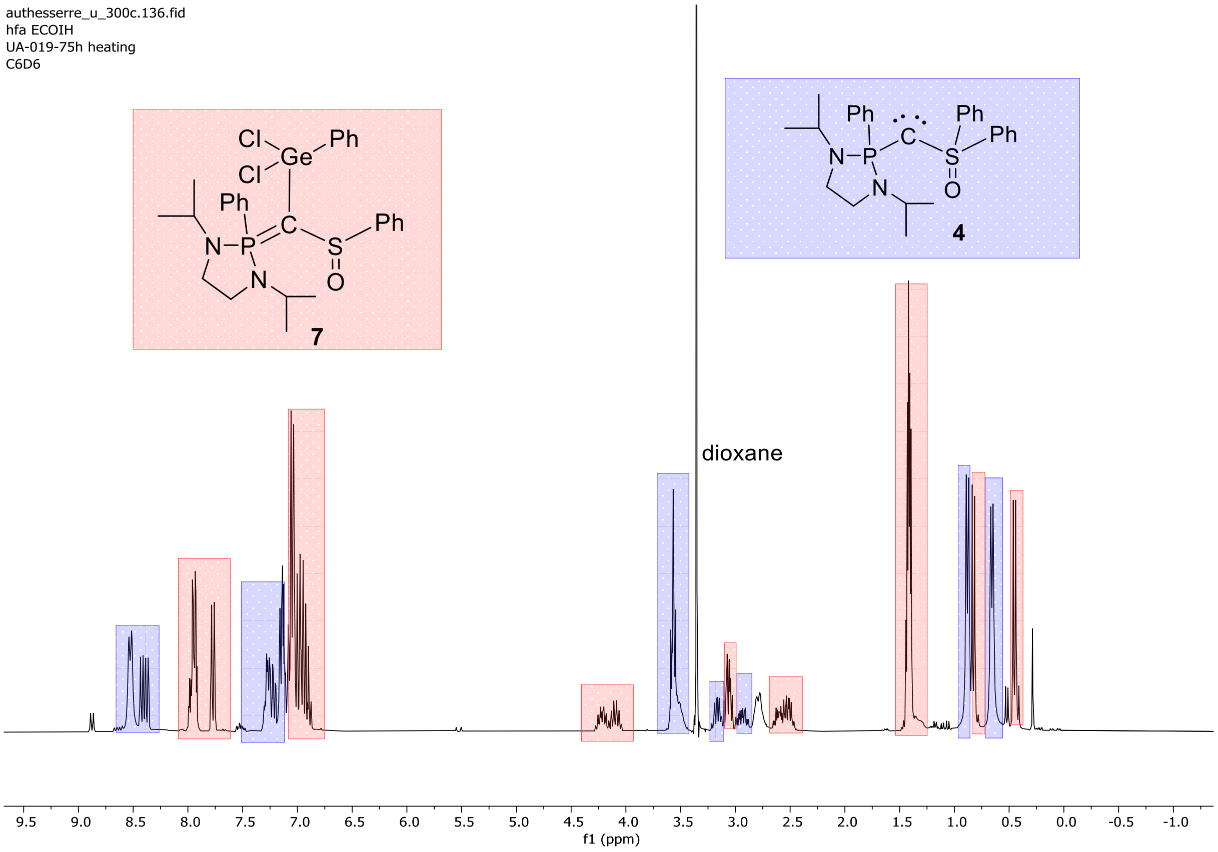

Figure S11: ^{1}H NMR (300 MHz, C_6D_6)

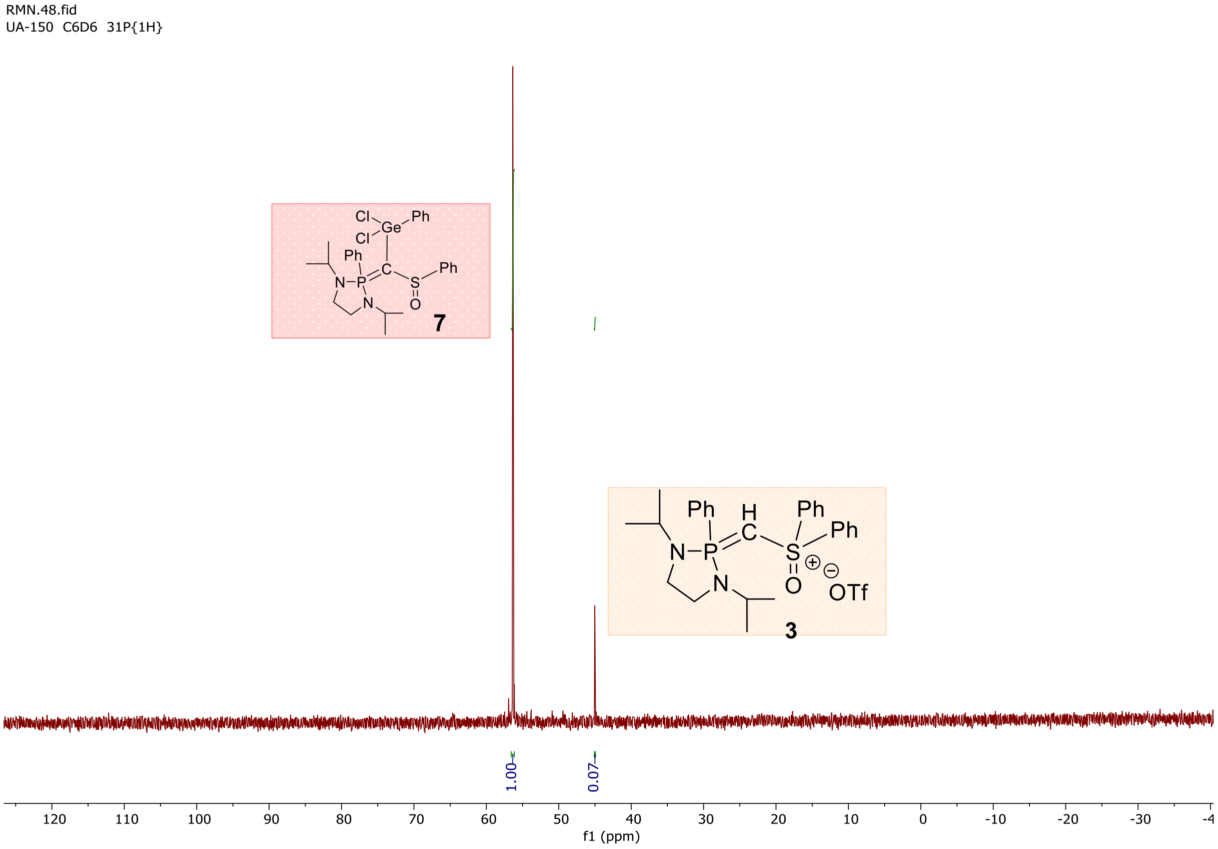

Figure S12: ^{31}P{^{1}H} NMR (300 MHz, C_6D_6) Migration reaction using 10 mol% of **4**, that get reprotonated into **3** at the end of the heating.

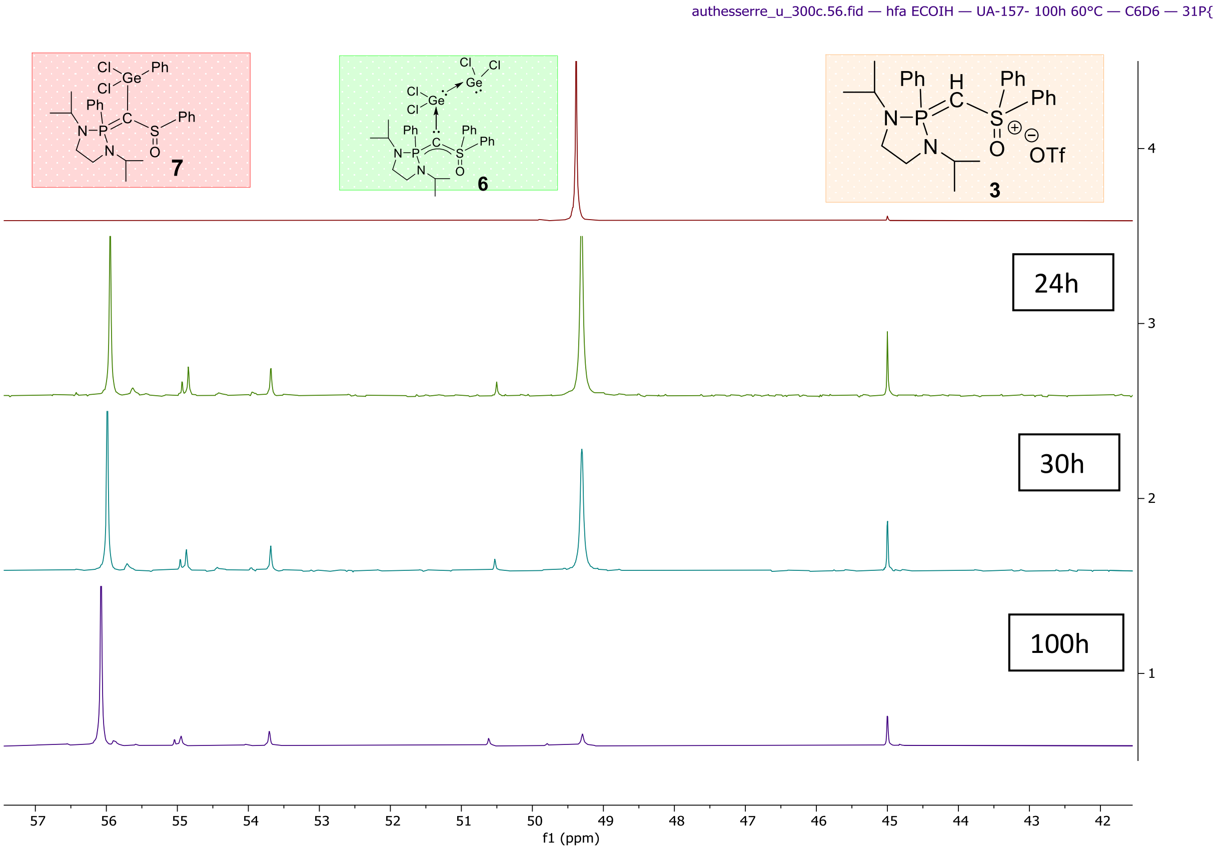

Figure S13: Monitoring $^{31}P\{^{1}H\}$ NMR (300 MHz, C_6D_6) Migration reaction using 15 mol% of Et_3N.

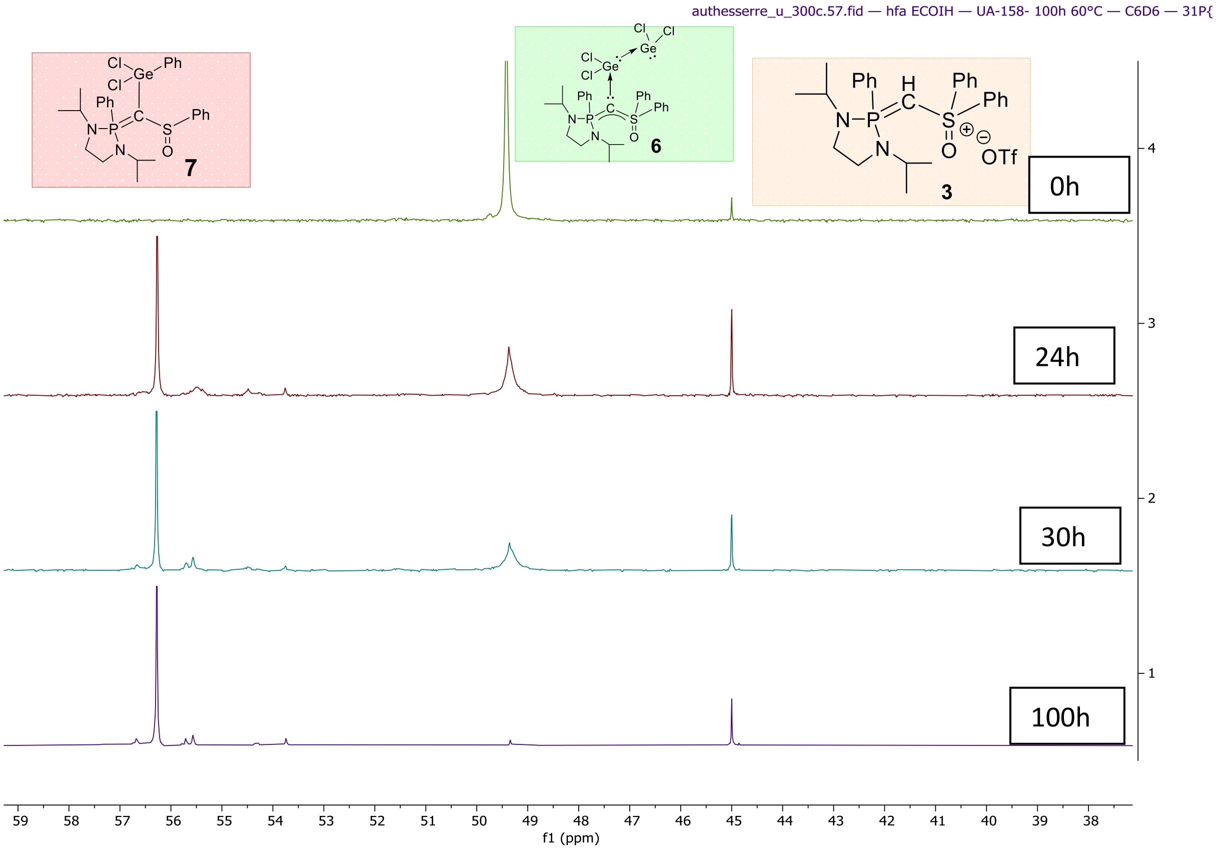

Figure S14: Monitoring $^{31}P\{^{1}H\}$ NMR (300 MHz, C_6D_6) Migration reaction using 15 mol% of DMAP.

X-ray analysis

The data of the structures for **5**, **6** and **7** were collected at 193 K on a Bruker-AXS APEX II CCD Quazar diffractometer (**7**) equipped with a 30 W air-cooled microfocus source or on a Brucker-AXS D8-Venture diffractometer (**5** and **6**) equipped with a CMOS Area detector with MoKα radiation (wavelength = 0.71073 Å) by using phi- and omega-scans. The data were integrated with SAINT, and an empirical absorption correction with SADABS was applied [44]. The structures were solved using an intrinsic phasing method (ShelXT) [45] and refined using the least–squares method on F^2 (ShelXL-2014) [46]. All non-H atoms were treated anisotropically. All H atoms attached to C atoms were fixed geometrically and treated as riding on their parent atoms with C-H = 0.95 Å (aromatic), 0.98 Å (CH_3), 0.99 Å (CH_2) or 1.0 Å (CH) with $U_{iso}(H) = 1.2U_{eq}(CH, CH_2)$ or $U_{iso}(H) = 1.5U_{eq}(CH_3)$. Supplementary crystallographic data for CCDC-2068304 (**5**), CCDC-2068305 (**6**), CCDC-2068306 (**7**) can be obtained free of charge from The Cambridge Crystallographic Data Centre via https://www.ccdc.cam.ac.uk/structures/.

The details of data collection and crystal structures refinement are summarized in Table 1.

Table S1. Crystallographic data for the compounds **5**, **6** and **7**

**Compound**	**5**	**6**	**7**
Chemical formula	$C_{27}H_{33}Cl_2GeN_2OPS$	$C_{27}H_{33}Cl_4Ge_2N_2OPS$	$C_{27}H_{33}Cl_2Ge N_2OPS$
M_r	608.07	751.56	608.07
Crystal system	Orthorhombic	Monoclinic	Monoclinic
Space group	$P2_12_12_1$	$P2_1$/n	$P2_1$/n
a [Å]	10.3934(6)	11.2219(5)	9.0496(12)
b [Å]	15.3160(9)	18.1701(7)	14.5792(16)
c [Å]	18.0563(11)	15.7026(6)	22.036(3)
α [°]	90	90	90
β [°]	90	93.4488(15)	90.653(4)
γ [°]	90	90	90
V [$Å^3$]	2874.3(3)	3196.0(2)	2907.2(6)
Z	4	4	4
ρ [g cm^{-3}]	1.405	1.562	1.389
$\mu(Mo_{K\alpha})$ [mm^{-1}]	1.403	2.355	1.387
Reflections collected	113982	125271	72609
Independent reflections	7146 R(int)=0.0627	9769 R(int)=0.0497	5915 R(int)=0.1491
Data/ restraints/ parameters	7146/0/320	9769/0/347	5915/0/320
Crystal size [mm^3]	0.460x0.200x0.160	0.380×0.200×0.120	0.100×0.060×0.020
GOOF on F^2	1.070	1.029	1.030
R (I > 2σ(I))	0.0291	0.0306	0.0456
wR2 (all data)	0.0561	0.0741	0.0916
Largest difference peak and hole, [e $Å^{-3}$]	0.337 and -0.267	0.666 and -0.613	0.343 and -0.453
CCDC number	2068304	2068305	2068306

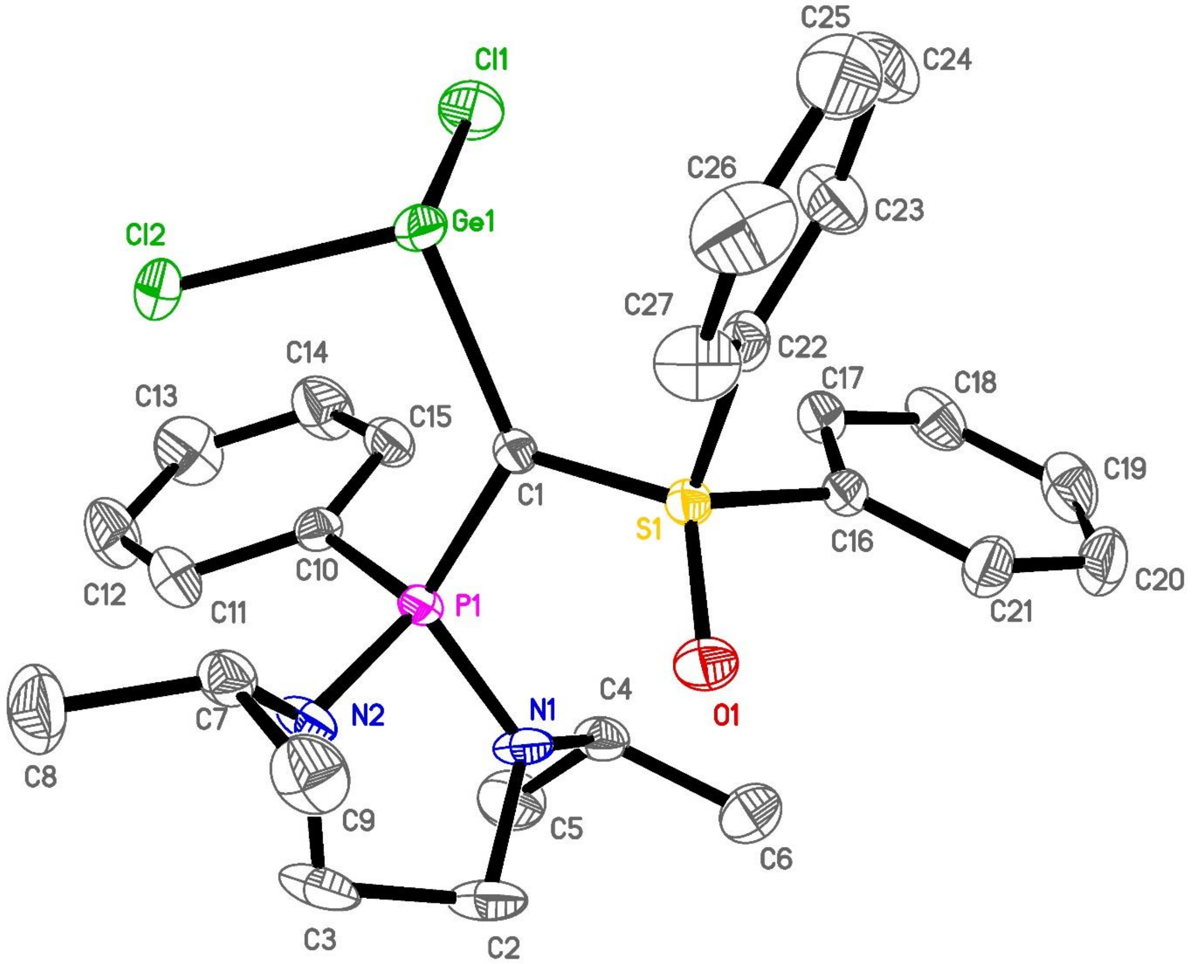

Figure S15: Molecular view of **5**. Thermal ellipsoids set at 50 % probability. H atoms are omitted for clarity. Selected bond lengths [Å]:

C1-Ge1 2.071(2), C1-P1 1.725(2), C1-S1 1.650(2), P1-C10 1.802(3), S1-O1 1.457(2), Ge1-Cl2 2.299(1), Ge1-Cl1 2.331(1), P1-N1 1.649(2), P1-N2 1.648(2), S1-C22 1.780(3), S1-C16 1.784(3). P1-C1-S1 113.73(14), P1-C1-Ge1 128.12(13), Ge1-C1-S1 115.65(13), C1-Ge1-Cl2 96.82(7), Cl2-Ge1-Cl1 95.37(3), Cl1-Ge1-C1 98.81(7).

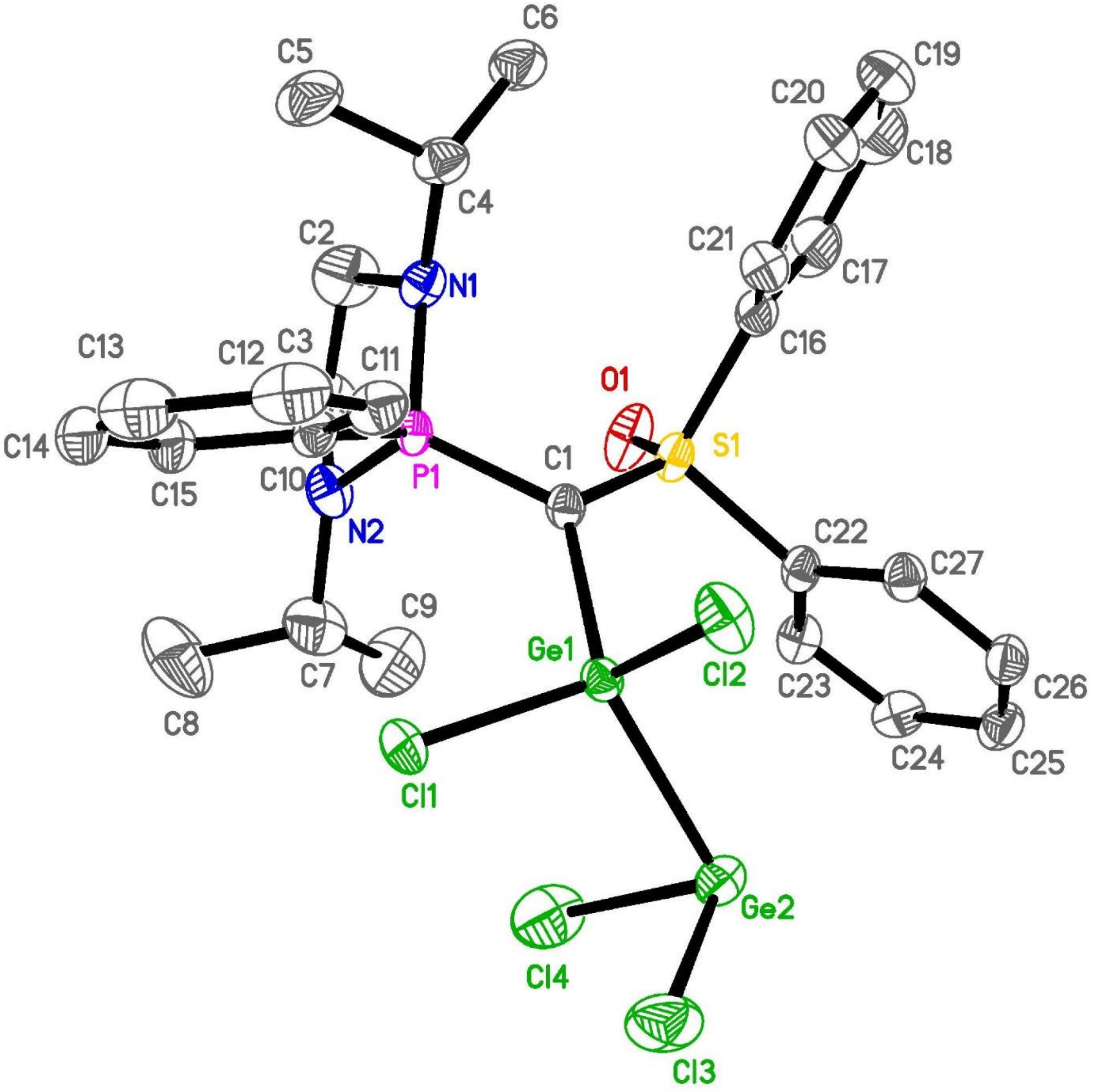

Figure S16: Molecular view of **6**. Thermal ellipsoids set at 50 % probability. H are omitted for clarity. Selected bond lengths [Å] and angles [°]: C1-Ge1 1.980(2), C1-P1 1.748(2), C1-S1 1.665(2), Ge1-Ge2 2.582(1), Ge1-Cl1 2.185(1), Ge1-Cl2 2.245(1), Ge2-Cl4 2.267(1), Ge2-Cl3 2.275(1), P1-C10 1.792(2), S1-O1 1.455(1), S1-C22 1.778(2), S1-C16 1.782(2), P1-C1-S1 112.40(10), P1-C1-Ge1 127.01(9), Ge1-C1-S1 117.30(9), C1-Ge1-Cl1 105.44(5), Cl1-Ge1-Cl2 101.33(2), Cl2-Ge1-Ge2 107.25(2), Cl1-Ge1-Ge2 109.25(2), Ge1-Ge2-Cl4 92.09(2), Cl4-Ge2-Cl3 98.84(3), Ge1-Ge2-Cl3 89.41(2).

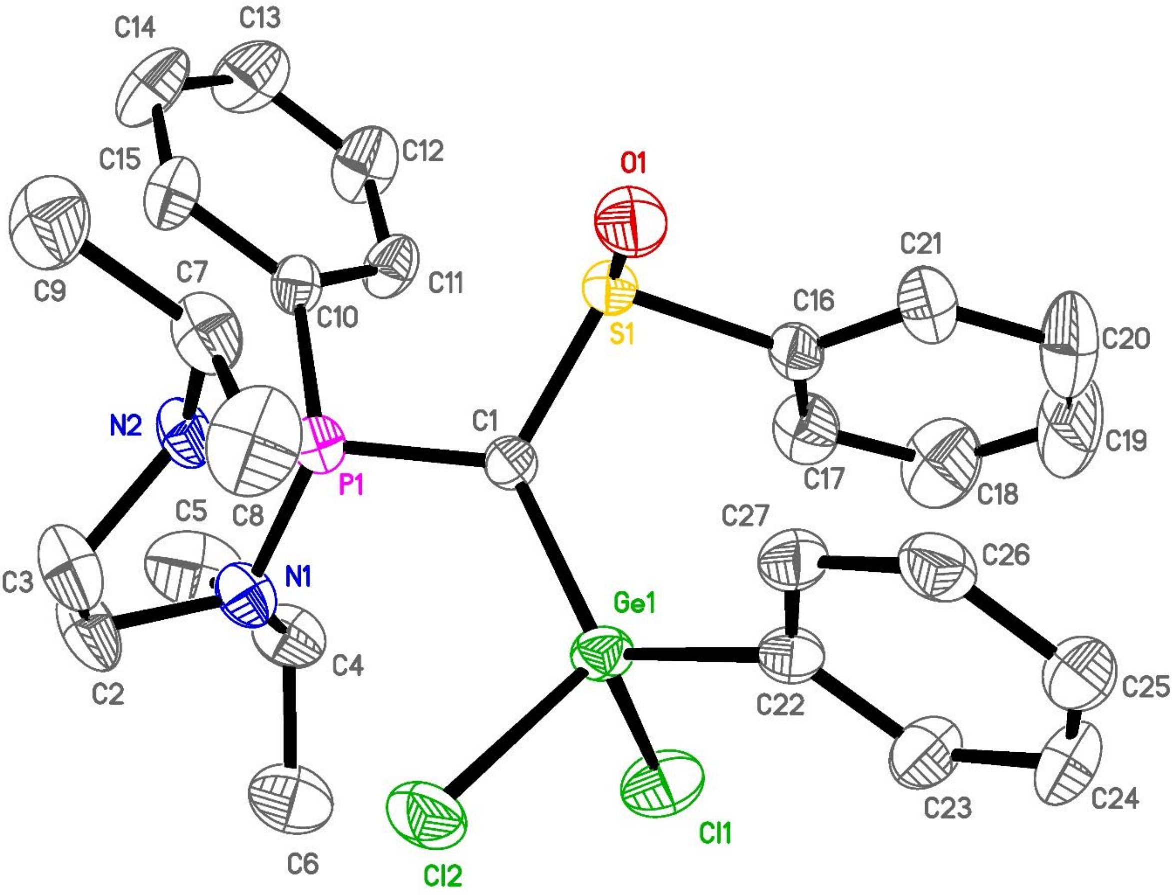

Figure S17: Molecular view of **7**. Thermal ellipsoids set at 50 % probability. H atoms are omitted for clarity. Selected bond lengths [Å] and angles [°]: P1-C1 1.707(3), C1-S1 1.744(3), C1-Ge1 1.891(3), Ge1-C22 1.927(3), Ge1-C22 1.927(3), Ge1-Cl2 2.172(1), Ge1-Cl1 2.173(1), P1-C10 1.805(3), S1-O1 1.499(2), S1-C16 1.800(4), P1-C1-S1 114.48(18), S1-C1-Ge1 120.89(17), Ge1-C1-P1 122.37(19), C1-Ge1-C22 119.52(14), C1-Ge1-Cl2 111.27(10), C22-Ge1-Cl2 104.77(10), C1-Ge1-Cl1 111.72(10), C22-Ge1-Cl1 106.65(11), Cl2-Ge1-Cl1 101.15(4), N1-P1-N2 94.89(15), N1-P1-C1 113.16(16), N2-P1-C1, 120.48(15), N1-P1-C10 111.60(15), N2-P1-C10 108.20(16), C1-P1-C10 107.98(16), O1-S1-C1 112.64(15), O1-S1-C16 105.28(16), C1-S1-C16 100.04(16), C4-N1-C2 119.6(3), C4-N1-P1 122.2(2), C2-N1-P1 111.5(2), C3-N2-C7 120.0(3), C3-N2-P1 113.4(2), C7-N2-P1 122.5(2).
