# Supplementary material for: Complexes of Dichlorogermylene with Phosphine/Sulfoxide-Supported Carbone as Ligand
Source: Molecules. 2021 Apr 1;26(7):2005. doi: 10.3390/molecules26072005 (PMC8037485; doi:10.3390/molecules26072005)

checkCIF/PLATON report

Structure factors have been supplied for datablock(s) 5, 6, 7

THIS REPORT IS FOR GUIDANCE ONLY. IF USED AS PART OF A REVIEW PROCEDURE FOR PUBLICATION, IT SHOULD NOT REPLACE THE EXPERTISE OF AN EXPERIENCED CRYSTALLOGRAPHIC REFEREE.

No syntax errors found. CIF dictionary Interpreting this report

Datablock: 5

```
Bond precision:  C-C = 0.0042 A          Wavelength=0.71073

Cell:            a=10.3934(6)     b=15.3160(9)     c=18.0563(11)
                 alpha=90         beta=90          gamma=90
Temperature:     193 K

                Calculated                  Reported
Volume          2874.3(3)                   2874.3(3)
Space group     P 21 21 21                  P 21 21 21
Hall group      P 2ac 2ab                   P 2ac 2ab
Moiety formula  C27 H33 Cl2 Ge N2 O P S     ?
Sum formula     C27 H33 Cl2 Ge N2 O P S     C27 H33 Cl2 Ge N2 O P S
Mr              608.09                      608.07
Dx,g cm-3       1.405                       1.405
Z               4                           4
Mu (mm-1)       1.403                       1.403
F000            1256.0                      1256.0
F000'           1258.97
h,k,lmax        13,20,24                    13,20,24
Nref            7158[ 4001]                 7146
Tmin,Tmax       0.721,0.799                 0.616,0.746
Tmin'           0.519

Correction method= # Reported T Limits: Tmin=0.616 Tmax=0.746
AbsCorr = MULTI-SCAN

Data completeness= 1.79/1.00      Theta(max)= 28.319

R(reflections)= 0.0291( 6501)     wR2(reflections)= 0.0561( 7146)

S = 1.070                  Npar= 320
```

The following ALERTS were generated. Each ALERT has the format
test-name_ALERT_alert-type_alert-level.
Click on the hyperlinks for more details of the test.

Alert level C

```
PLAT910_ALERT_3_C Missing # of FCF Reflection(s) Below Theta(Min).         8 Note
```

Alert level G

```
PLAT883_ALERT_1_G No Info/Value for _atom_sites_solution_primary .     Please Do !
PLAT913_ALERT_3_G Missing # of Very Strong Reflections in FCF ....         1 Note
PLAT978_ALERT_2_G Number C-C Bonds with Positive Residual Density.         7 Info
```

```
   0 ALERT level A = Most likely a serious problem - resolve or explain
   0 ALERT level B = A potentially serious problem, consider carefully
   1 ALERT level C = Check. Ensure it is not caused by an omission or oversight
   3 ALERT level G = General information/check it is not something unexpected

   1 ALERT type 1 CIF construction/syntax error, inconsistent or missing data
   1 ALERT type 2 Indicator that the structure model may be wrong or deficient
   2 ALERT type 3 Indicator that the structure quality may be low
   0 ALERT type 4 Improvement, methodology, query or suggestion
   0 ALERT type 5 Informative message, check
```

Datablock: 6

```
Bond precision:  C-C = 0.0033 A          Wavelength=0.71073

Cell:           a=11.2219(5)    b=18.1701(7)        c=15.7026(6)
                alpha=90        beta=93.4488(15)    gamma=90
Temperature:    193 K

                Calculated                    Reported
Volume          3196.0(2)                     3196.0(2)
Space group     P 21/n                        P 21/n
Hall group      -P 2yn                        -P 2yn
Moiety formula  C27 H33 Cl4 Ge2 N2 O P S     ?
Sum formula     C27 H33 Cl4 Ge2 N2 O P S     C27 H33 Cl4 Ge2 N2 O P S
Mr              751.60                        751.56
Dx,g cm-3       1.562                         1.562
Z               4                             4
Mu (mm-1)       2.355                         2.355
F000            1520.0                        1520.0
F000’           1524.76
h,k,lmax        16,25,22                      16,25,22
Nref            9811                          9769
Tmin,Tmax       0.574,0.754                   0.651,0.746
Tmin’           0.405

Correction method= # Reported T Limits: Tmin=0.651 Tmax=0.746
AbsCorr = MULTI-SCAN
```

```
Data completeness= 0.996          Theta(max)= 30.561

R(reflections)= 0.0306( 7740)     wR2(reflections)= 0.0741( 9769)

S = 1.029                   Npar= 347
```

```
The following ALERTS were generated. Each ALERT has the format
       test-name_ALERT_alert-type_alert-level.
Click on the hyperlinks for more details of the test.
```

Alert level B

```
PLAT230_ALERT_2_B Hirshfeld Test Diff for    Ge1      --Ge2      .       14.1 s.u.
PLAT910_ALERT_3_B Missing # of FCF Reflection(s) Below Theta(Min).         11 Note
```

Alert level C

```
PLAT220_ALERT_2_C NonSolvent   Resd 1  C   Ueq(max)/Ueq(min) Range        3.6 Ratio
```

Alert level G

```
PLAT432_ALERT_2_G Short Inter X...Y Contact  O1       ..C24             2.96 Ang.
                                               1-x,1-y,1-z  =      3_666 Check
PLAT883_ALERT_1_G No Info/Value for _atom_sites_solution_primary .     Please Do !
PLAT912_ALERT_4_G Missing # of FCF Reflections Above STh/L=  0.600         31 Note
PLAT913_ALERT_3_G Missing # of Very Strong Reflections in FCF ....          2 Note
PLAT978_ALERT_2_G Number C-C Bonds with Positive Residual Density.         10 Info
```

```
   0 ALERT level A = Most likely a serious problem - resolve or explain
   2 ALERT level B = A potentially serious problem, consider carefully
   1 ALERT level C = Check. Ensure it is not caused by an omission or oversight
   5 ALERT level G = General information/check it is not something unexpected

   1 ALERT type 1 CIF construction/syntax error, inconsistent or missing data
   4 ALERT type 2 Indicator that the structure model may be wrong or deficient
   2 ALERT type 3 Indicator that the structure quality may be low
   1 ALERT type 4 Improvement, methodology, query or suggestion
   0 ALERT type 5 Informative message, check
```

Datablock: 7

```
Bond precision:   C-C = 0.0055 A           Wavelength=0.71073

Cell:             a=9.0496(12)      b=14.5792(16)       c=22.036(3)
                  alpha=90          beta=90.653(4)      gamma=90
Temperature:      193 K
```

	Calculated	Reported
Volume	2907.2(6)	2907.2(6)
Space group	P 21/n	P 21/n
Hall group	-P 2yn	-P 2yn
Moiety formula	C27 H33 Cl2 Ge N2 O P S	?
Sum formula	C27 H33 Cl2 Ge N2 O P S	C27 H33 Cl2 Ge N2 O P S
Mr	608.09	608.07
Dx,g cm-3	1.389	1.389
Z	4	4
Mu (mm-1)	1.387	1.387
F000	1256.0	1256.0
F000’	1258.97	
h,k,lmax	11,18,27	11,18,27
Nref	5938	5915
Tmin,Tmax	0.905,0.973	0.672,0.719
Tmin’	0.870	

Correction method= # Reported T Limits: Tmin=0.672 Tmax=0.719
AbsCorr = MULTI-SCAN

Data completeness= 0.996   Theta(max)= 26.371

R(reflections)= 0.0456(3770)   wR2(reflections)= 0.0916(5915)

S = 1.030   Npar= 320

The following ALERTS were generated. Each ALERT has the format
test-name_ALERT_alert-type_alert-level.
Click on the hyperlinks for more details of the test.

Alert level B

PLAT910_ALERT_3_B Missing # of FCF Reflection(s) Below Theta(Min).   11 Note

---

Alert level C

RINTA01_ALERT_3_C The value of Rint is greater than 0.12
Rint given 0.149
PLAT911_ALERT_3_C Missing FCF Refl Between Thmin & STh/L= 0.600   13 Report

---

Alert level G

PLAT020_ALERT_3_G The Value of Rint is Greater Than 0.12 ....   0.149 Report
PLAT883_ALERT_1_G No Info/Value for _atom_sites_solution_primary .   Please Do !
PLAT913_ALERT_3_G Missing # of Very Strong Reflections in FCF ....   2 Note
PLAT933_ALERT_2_G Number of OMIT Records in Embedded .res File ...   16 Note
PLAT978_ALERT_2_G Number C-C Bonds with Positive Residual Density.   3 Info

---

0 **ALERT level A** = Most likely a serious problem - resolve or explain
1 **ALERT level B** = A potentially serious problem, consider carefully
2 **ALERT level C** = Check. Ensure it is not caused by an omission or oversight
5 **ALERT level G** = General information/check it is not something unexpected

```
1 ALERT type 1 CIF construction/syntax error, inconsistent or missing data
2 ALERT type 2 Indicator that the structure model may be wrong or deficient
5 ALERT type 3 Indicator that the structure quality may be low
0 ALERT type 4 Improvement, methodology, query or suggestion
0 ALERT type 5 Informative message, check
```

It is advisable to attempt to resolve as many as possible of the alerts in all categories. Often the minor alerts point to easily fixed oversights, errors and omissions in your CIF or refinement strategy, so attention to these fine details can be worthwhile. In order to resolve some of the more serious problems it may be necessary to carry out additional measurements or structure refinements. However, the purpose of your study may justify the reported deviations and the more serious of these should normally be commented upon in the discussion or experimental section of a paper or in the "special_details" fields of the CIF. checkCIF was carefully designed to identify outliers and unusual parameters, but every test has its limitations and alerts that are not important in a particular case may appear. Conversely, the absence of alerts does not guarantee there are no aspects of the results needing attention. It is up to the individual to critically assess their own results and, if necessary, seek expert advice.

Publication of your CIF in IUCr journals

A basic structural check has been run on your CIF. These basic checks will be run on all CIFs submitted for publication in IUCr journals (*Acta Crystallographica*, *Journal of Applied Crystallography*, *Journal of Synchrotron Radiation*); however, if you intend to submit to *Acta Crystallographica Section C* or *E* or *IUCrData*, you should make sure that full publication checks are run on the final version of your CIF prior to submission.

Publication of your CIF in other journals

Please refer to the *Notes for Authors* of the relevant journal for any special instructions relating to CIF submission.

PLATON version of 05/12/2020; check.def file version of 05/12/2020

Datablock 5 - ellipsoid plot

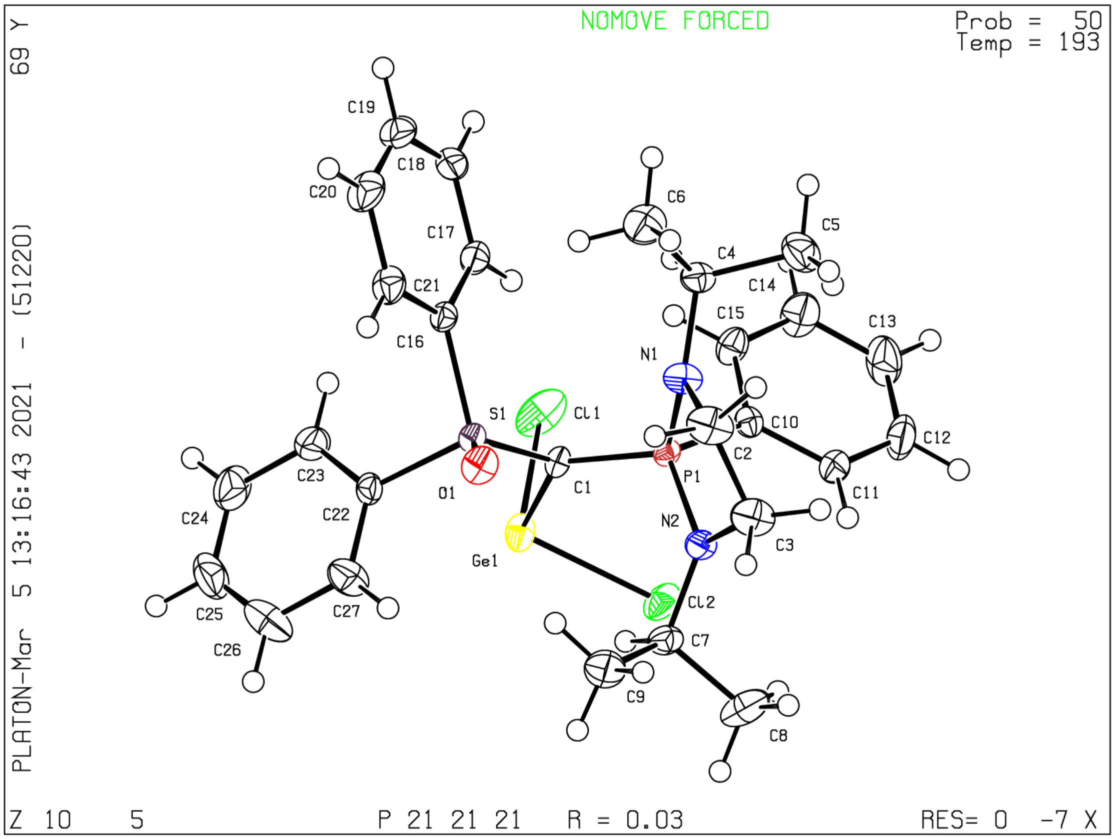

Datablock 6 - ellipsoid plot

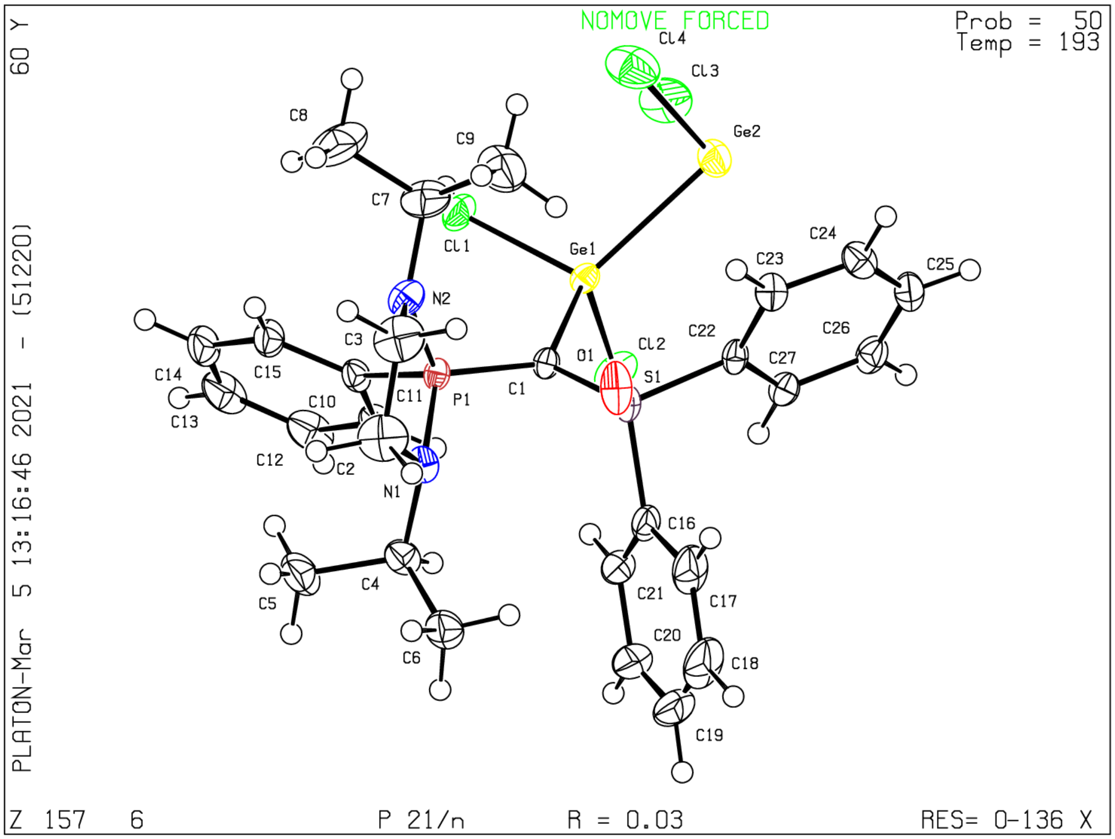

Datablock 7 - ellipsoid plot

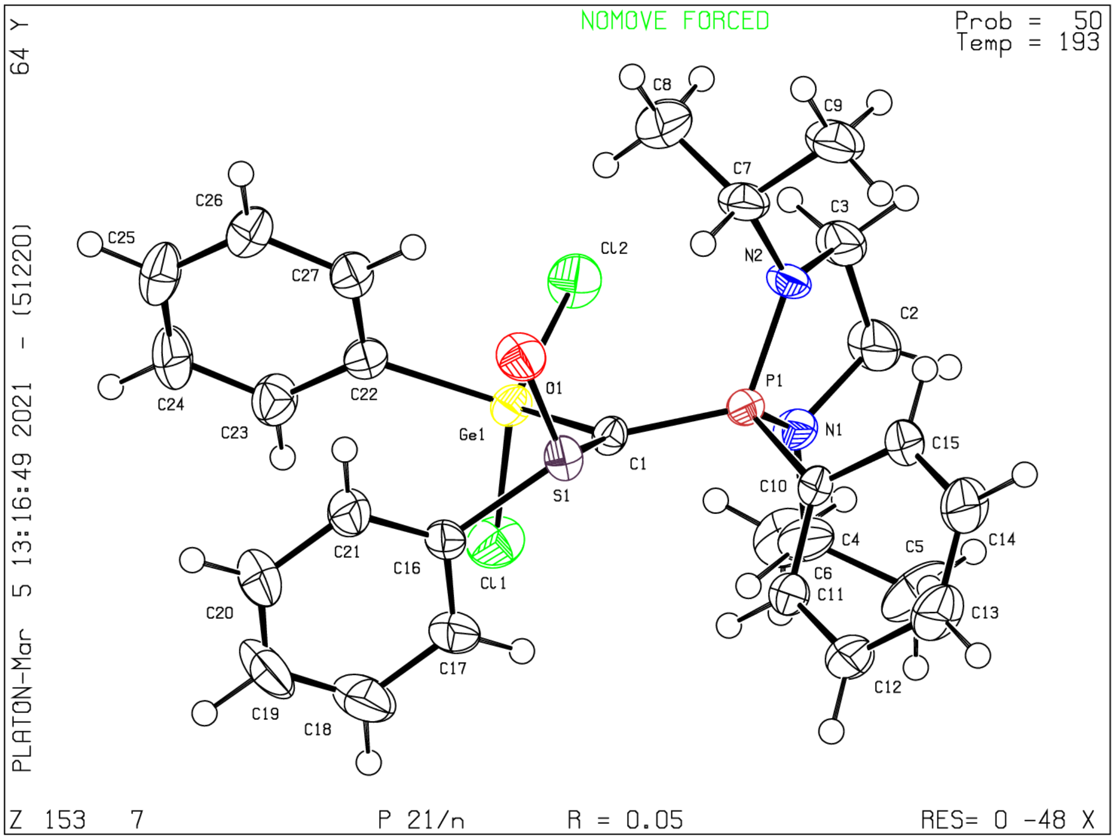

Supplement: Supplementary file 1 [file molecules-26-02005-s001.zip › checkcif_global.pdf]
